# Targeting AKT induced Ferroptosis through FTO/YTHDF2-dependent GPX4 m6A methylation up-regulating and degradating in colorectal cancer

**DOI:** 10.1038/s41420-023-01746-x

**Published:** 2023-12-15

**Authors:** Ge Zhang, Wunan Mi, Chuyue Wang, Jiehan Li, Yizheng Zhang, Nannan Liu, Meimei Jiang, Guiyun Jia, Feng Wang, Ge Yang, Lingling Zhang, Jiangang Wang, Yang Fu, Yingjie Zhang

**Affiliations:** 1 Department of Health Management, The Third Xiangya Hospital, Central South University, Changsha, China; 2 Department of Gastrointestinal Surgery, The First Affiliated Hospital of Zhengzhou University, Zhengzhou, 450052 China; 3 School of Biomedical Sciences, Hunan University, Changsha, 410082 China; 4 Department of Surgery, Erasmus MC Transplant Institute, Erasmus MC University Medical Center, Rotterdam, The Netherlands; 5 Department of Gastroenterology, The Tenth People’s Hospital of Shanghai, Tongji University, Shanghai, 200072 China; 6 Department of Ophthalmology, The First Affiliated Hospital of Zhengzhou University, Zhengzhou, 450052 China; 7 Department of Laboratory Medicine, The Third Xiangya Hospital, Central South University, Changsha, 410013 China; 8 Department of Gastroenterology, Huadong Hospital, Shanghai Medical College, Fudan University, Shanghai, 200040 PR China

**Keywords:** Cancer epigenetics, Chemotherapy

## Abstract

Ferroptosis is a new type of iron-dependent programmed cell death induced by lipid peroxidation. However, the underlying mechanisms and function in tumor therapy still remain undisclosed especially in post-transcription regulation. Here, we found that targeting AKT significantly induced GPX4 dependent ferroptosis and suppressed colorectal cancer growth both in vitro and in vivo. During this process, demethylase FTO was downregulated, which increased the m6A methylation level of GPX4, subsequently recognized by YTHDF2 and degraded. Prediction results showed that there are three potential methylated sites (193/647/766), and 193 site was identified as the right one, which was demethylated by FTO and read by YTHDF2. In parallel, AKT inhibition caused the accumulation of ROS which had a negative feedback on GPX4 expression. In addition, protective autophagy was initiated by MK2206 stimulation, while blocking autophagy further increased ferroptosis and markedly enhanced the anti-tumor activity of MK2206. In a word, inhibiting AKT activated ferroptosis through FTO/YTHDF2/GPX4 axis to suppress colon cancer progression, which raised FTO/GPX4 as potential biomarkers and targets in colorectal cancer therapy.

## Introduction

Colorectal cancer is the third malignant tumor in the world in terms of morbidity and mortality [1]. Globally, there are >1.8 million new diagnostic CRC patients and 850,000 CRC-related deaths every year [2]. The most common treatment option for CRC is surgery, but there is still a high percentage of postoperative recurrences after suegery [3]. Consequently, it is urgent to develop new treatment strategies to hinder tumor progression thus improving the prognosis of patients with CRC.

PI3K-AKT signaling pathway is a vital regulator in diverse biological processes, such as cell growth, proliferation and metabolism [4]. Activation of AKT promotes cell division and proliferation in various cancers, which also contributes to block drug resistance and cell death. Conversely, block of AKT reduces cell survival, which is also regarded as a potential therapeutic strategy to strengthen the drug antitumor effect, especially in the case of drug resistance [5]. In the current reports, AKT is intimately related to the occurrence of various cell death modes, such as pyroptosis, apoptosis, autophagy, parthanatos and ferroptosis [6], but the mechanism of how it regulates ferroptosis still needs to be perfected.

Ferroptosis is an iron-dependent programmed cell death modes recently discovered, which is induced by peroxidized lipids [7]. Its morphological characteristics, action mode and molecular mechanisms of ferroptosis are completely distinct from other types of programmed death [8], such as apoptosis, necroptosis, pyroptosis and cuprotosis [9–11]. Key features of ferroptosis include iron-dependent lipid peroxidation, glutathione peroxidase 4 (GPX4) degradation, and condensed mitochondrial membrane densities [12]. Recently, increasing evidence suggests that the regulation of ferroptosis requires through autophagy [13, 14], but it is not really clear.

m6A methylation, the most common post-transcriptional modification on mRNA [15], is regulated by “writer (methylation executor)”, “eraser (demethylation executor)” and “reader (m6A site recognizer)“ [16]. “Writer” is a methyltransferase, which is responsible for mediating the methylation process, such as METTL3, METTL14, METTL16, WTAP and KIAA1429 [17, 18]. While “eraser” is a demethylase and facilitates the reversible process of m6A demethylation, like FTO and ALKBH5 [19]. “readers” regulated the target mRNA after m6A modification, including YTH domain family and IGF2BP family [20, 21], such as YTHDF1-3 and YTHDC1-2.

In our previous study, we found that targeting ERK could induce protective autophagy in colorectal cancer [22]. But it is not fully clear whether protective autophagy also happens in ferroptosis. In addition, the function of post-transcriptional regulation like m6A RNA modification in ferroptosis almost rarely explored. In this study, we confirmed a novel post-transcriptional regulatory mechanism of ferroptosis that blocking AKT decreased FTO thus reduced GPX4 expression in a YTHDF2-dependent manner and confirmed the 193 site is a definitely methylated site on GPX4 mRNA. In addition, we also demonstrated the protective autophagy in ferroptosis.

## Results

### Targeting AKT inhibited the proliferation of colorectal cancer cells

**Fig. 1:**
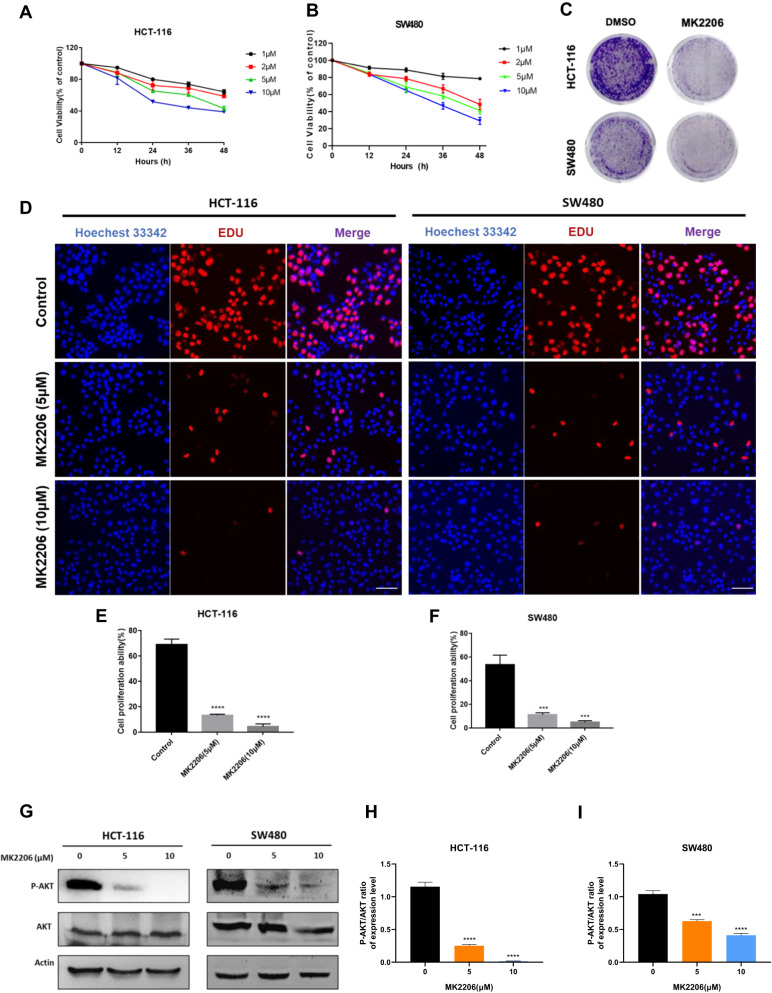
MK2206 inhibited cell proliferation in CRC cells. **A**, **B** HCT-116 and SW480 were incubated with different concentrations of MK2206 for 12 h, 24 h, 36 h and 48 h, and cell viability was subsequently detected by the CCK-8 assay subsequently. **C** Cell colony formation of HCT-116 and SW480 were treated with 10 μM of MK2206 for 2 weeks. **D**–**F** EDU was used to detect cell proliferation after treatment with 5 μM and 10 μM MK2206 [scale bar, 100 μm]. **G**–**I** HCT-116 and SW480 were treated with 0, 5 and 10 μM MK2206 for 24 h, then the protein expression levels of P-AKT, AKT and β-Actin were detected by western blotting after 24 h.

In order to verify the important role of AKT pathway in the occurrence and development of CRC, MK2206, an AKT inhibitor, was used and determined the applicable concentration dose and time for the drug treatment in our study. Different doses of MK2206 (1, 2, 5 and 10 μM) was added in HCT-116 and SW480 cells, and cell viability was detected by CCK-8 assay after 0, 12, 24, 36 and 48 h of treatment. With the passage of dose and time, the cell proliferation ability decreased gradually in CRC cells (Fig. 1A, B). The reduction of cell colony formation was also observed after 10 μm MK2206 treatment of 3 days (Fig. 1C). In parallel, the EDU staining experiments gave us the similar results (Fig. 1D–F). Then, the phosphorylational levels of AKT (P-AKT) was detected and found effectively decreased after handling with MK2206 (Fig. 1G–I). As a direct downstream target of Akt, 4EBP1’s activity (P-4EBP1) was detected with P-AKT after treating with MK2206 (1, 2, 5, and 10 μM). The results showed that lower doses (1 or 2 uM) of MK2206 could inactivate 4EBP1, while for Akt inhibition, higher doses (5 or 10 uM) were needed (Fig. S1A–H). Together, these phenomena indicated that targeting AKT suppressed CRC growth and motivated us to investigate the mechanism behind.

### Targeting AKT induced ferroptosis

**Fig. 2:**
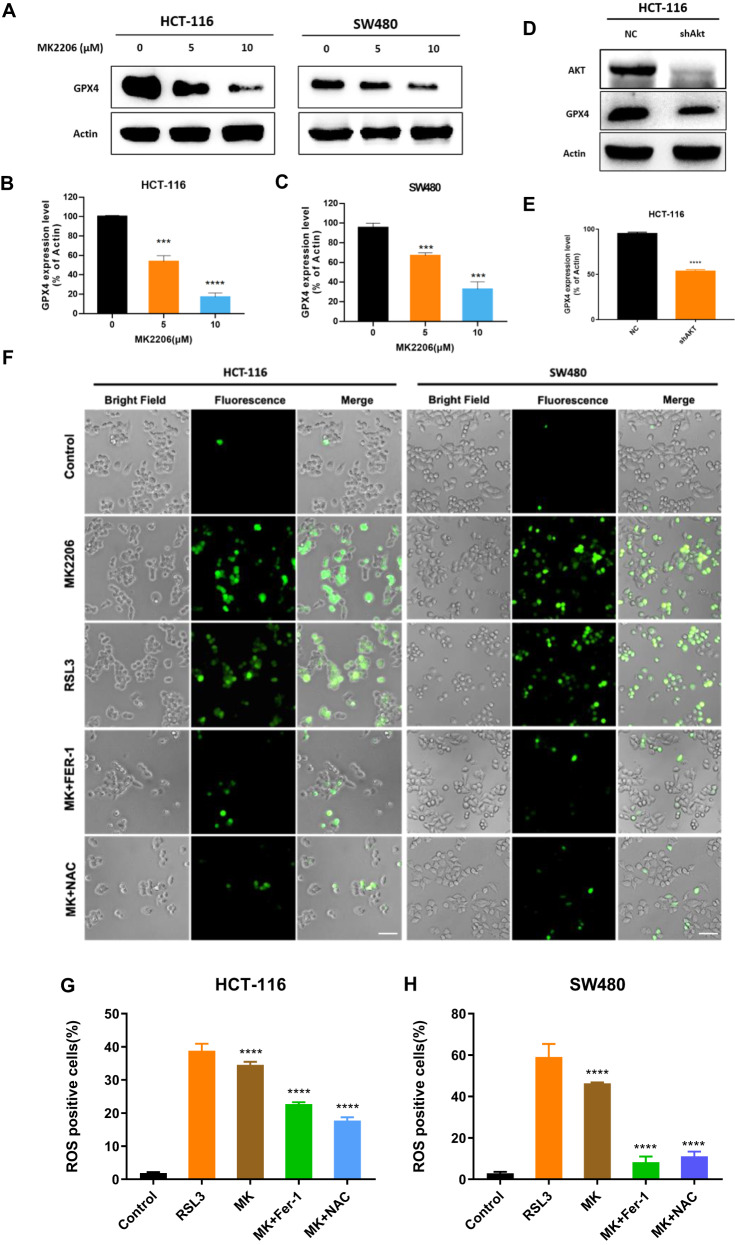
MK2206 induced ferroptosis in CRC cells. **A**–**C** HCT-116 and SW480 were cultured 24 h with 0, 5 and 10 μM of MK2206, then GPX4 and β-Actin expression levels were detected by western blotting. **D**, **E** The expression levels of AKT, GPX4 and β-Actin were detected after AKT knockdown. **F**–**H** ROS positive cells as assessed by DCFH-DA fluorescence in HCT-116 and SW480 were treated with MK2206(10 μM), RSL3(2 μM), MK2206 + FER-1(4 μM) and MK2206 + NAC(20 μM), and their representative images obtained by a fluorescence microscope[scale bar, 100 μm].

After treatment with 5 and 10 μM MK2206, the expression level of GPX4 was detected and found obviously decreased (Fig. 2A–C). In parallel, knocking down AKT gave us the similar results (Fig. 2D, E). As the downregulation of GPX4, ROS generation is also regarded as the marker of ferroptosis. So, in this study, the level of ROS was also detected, and RSL3 (ferroptosis inducer) treatment was set up as a positive control. As a result, ROS level rised after MK2206 treatment, as that of RSL3 (Fig. 2F–H), which further confirmed the induction of ferroptosis. However, this upregulation was markedly suppressed by Fer-1 (ferroptosis inhibitor) or NAC (ROS inhibitor), which was consistent with our expectation. Together, targeting AKT reduced the protein level of GPX4 and strengthened the level of ROS, which demonstrated the AKT inhibition induced the occurrence of ferroptosis via GPX4 decreasing.

### GPX4/ROS dependent ferroptosis played dominant role on inhibiting colon cancer cell proliferation

**Fig. 3:**
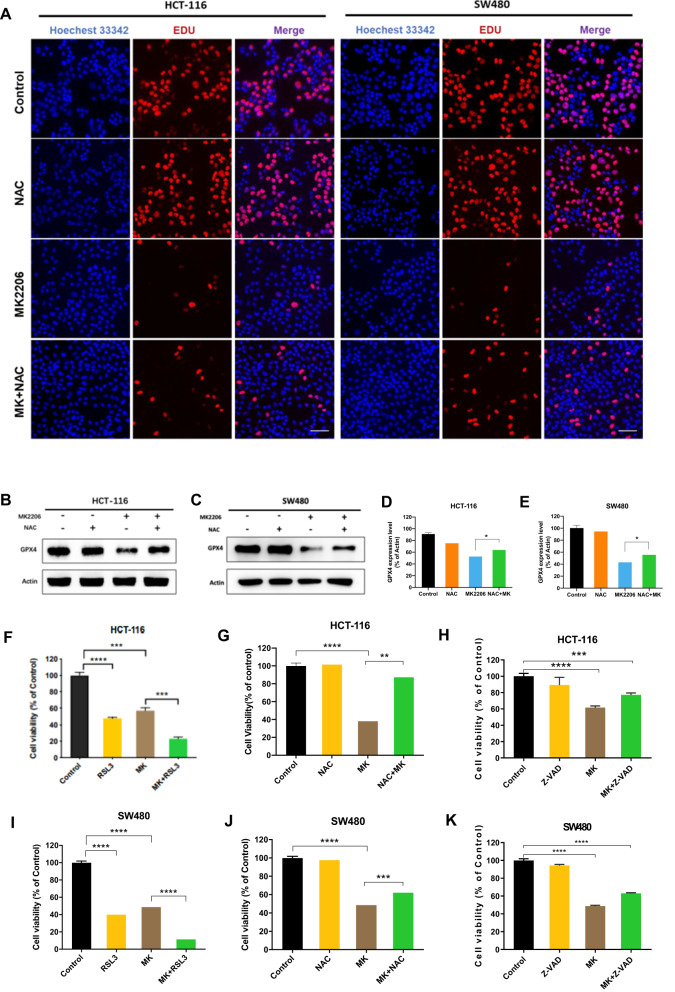
Cell death induced by MK2206 could be inhibited by NAC. **A** The EDU assay was applied to compare the cell proliferation ability in HCT-116 and SW480 that were treated with MK2206, NAC and MK2206 + NAC [scale bar, 100 μm]. **B**–**E** GPX4 expression levels were detected after treatment of HCT-116 and SW480 with MK2206, NAC and MK2206 + NAC. **F**–**K** MK2206 was used in combination with RSL3, NAC and Z-VAD to detect cell proliferation using the CCK-8 assay.

To further confirm that ferroptosis affects cell proliferation capacity, NAC was used on the basic of MK2206 treatment. EDU showed that the proliferation inhibition caused by MK2206 was partially restored by the co-treatment with NAC (Fig. 3A and S2A). Identical results were obtained from the CCK-8 detection (Fig. 3G, J). Next, the relationship between proliferation inhibition and ferroptosis was further investigated. As shown in Fig. 3B–E, GPX4 decrease caused by MK2206 was evidently restored by NAC. In addition, the combinational treatment of MK2206 and RSL3 had a synergistic effect to further suppress colon cancer cell growth (Fig. 3F, I). Interestingly, inhibiting apoptosis by using Z-VAD could not significantly rescued MK2206 caused cell growth suppression (Fig. 3H, K), which indicated that MK2206 induced ferroptosis and apoptosis simultaneously, while apoptosis played a minor role. Taken together, these above results demonstrated that targeting AKT induced GPX4/ROS dependent ferroptosis, which played a major role in suppressing colon cancer cell growth.

### Inhibition of AKT caused FTO down-regulation thus decreasing GPX4 expression

**Fig. 4:**
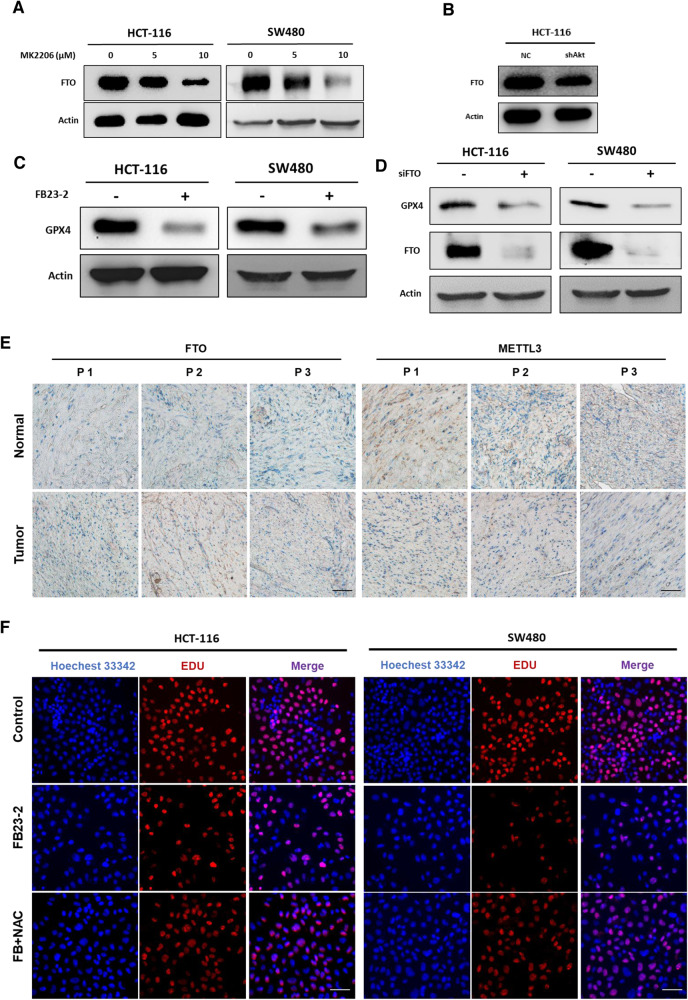
Inhibition of AKT causes downregulation of FTO. **A**, **B** FTO and β-Actin expression levels were detected after treatment of HCT-116 and SW480 with MK2206 or shAKT. **C** GPX4 and β-Actin expression levels were detected after treatment with FB23-2, a FTO inhibitor. **D** GPX4, FTO and β-Actin expression levels were detected after FTO knockdown. **E** FTO expression levels were verified in patient samples. **F** The EDU assay was used to compare the cell proliferation ability in HCT-116 and SW480 cells that were treated with FB23-2 and FB23-2 + NAC [scale bar, 100 μm].

It was discovered that FTO was significantly downregulated after AKT inhibition in this study (Fig. 4A and S3A), which was also confirmed after AKT knockdown (Fig. 4B and S3B). In order to further confirm whether the increased m6A methylation level caused by FTO downregulation regulates ferroptosis, FB23-2, an FTO inhibitor, was used and the expression level of GPX4 was significantly reduced by western blotting (Fig. 4C and S3C). In addition, to confirm whether the reduced expression of GPX4 was induced by FTO downregulation, CRC cells were treated with FTO siRNA and the expression of GPX4 was observed Then the relationship that FTO promotes GPX4 xpression was confirmed (Fig. 4D and S3D). In order to explore the level of methylation in CRC patients, FTO and METTL3 were stained by IHC in tumor tissues to discovered that FTO expression was higher in cancer tissues than in adjacent tissues, while METTL3 expression did not differ significantly between tumor and adjacent tissues (Fig. 4E). Furthermore, AKT, FTO and GPX4 were expressed at greater levels in cancer than adjacent tissues and found FTO and GPX4 are potential biomarkers in colorectal cancer (Fig. S4). And after inhibition of FTO, the cell proliferation was attenuated. However, this capacity could be in great part recovered following co-treatment with NAC (Fig. 4F and S3E). Together, these results show that m6A methylation modifications might play pivotal roles in the ferroptosis mediated by AKT inhibition.

### Blocking FTO increased the m6A methylation level of GPX4 which was recognized by YTHDF2 and subsequently degraded

**Fig. 5:**
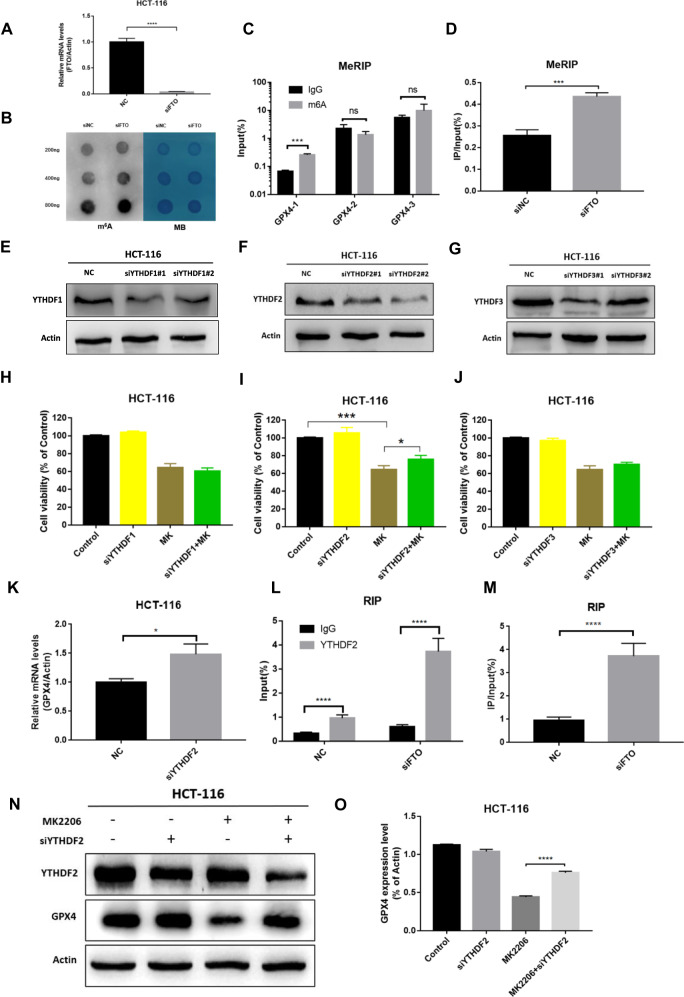
YTHDF2 regulated the expression of GPX4. **A**, **B** Dot blot were detected between NC and si FTO. **C**, **D** MeRIP-qPCR were detected after FTO knockdown. **E**–**G** The knockdown efficiency was tested by western blotting after YTHDF1, YTHDF2 and YTHDF3 knockdowns. **H**–**J** cell proliferation capacity was detected using the CCK-8 assay after YTHDF1, YTHDF2 and YTHDF3 knockdowns. **K**–**M** RIP were detected by YTHDF2 after FTO knockdown. **N**, **O** GPX4, YTHDF2 and β-Actin expression levels were detected after YTHDF2 knockdown and treatment with MK2206.

To confirm that m6A methylation is involved in the process of ferroptosis induced by AKT inhibition, the possible methylation modification sites 193, 647 and 766 on GPX4 mRNA were predicted by SRAMP (Fig. S5B) and the increased m6A methylation level was found by Dot blot assay (Fig. 5A, B). Then the different fragment primers were designed according to the different sites, and Me-RIP analysis finally confirmed that the 193 site on GPX4 mRNA was the m6A modification site (Fig. 5C, D). To further investigate the mechanisms underlying GPX4 m6A methylation regulation, YTHDF1/YTHDF2/YTHDF3 knocking-down experiments were firstly performed, and effective results were obtained (Fig. 5E–G and S5A). The CCK8 assay revealed that the decreased cell proliferation ability caused by MK2206 could be partially recovered by interfering YTHDF2 (Fig. 5H–J). YTHDF2 has been shown to detect m6A modification sites on mRNA and promote its degradation [23]. As a result, YTHDF2 was interfered, which lead to elevated GPX4 mRNA levels (Fig. 5K). To further explore the relationship of YTHDF2 and GPX4, RIP assay was designed, which confirmed the increased binding of YTHDF2 and GPX4 mRNA following FTO interfering (Fig. 5L, M). Furthermore, western blotting results indicated that the down-regulation of GPX4 induced by MK2206 could be restored after inhibiting the expression of YTHDF2, indicating that YTHDF2 could decrease the mRNA expression level of GPX4, thereby inhibiting the protein translation of GPX4 (Fig. 5N, O). In conclusion, FTO directly regulated the m6A modification of GPX4 mRNA on 193 site and YTHDF2 regulated AKT inhibition-induced ferroptosis by recognizing and degrading the m6A methylation modification sites on GPX4 mRNA.

### Inhibiting autophagy enhanced cell death induced by MK2206

**Fig. 6:**
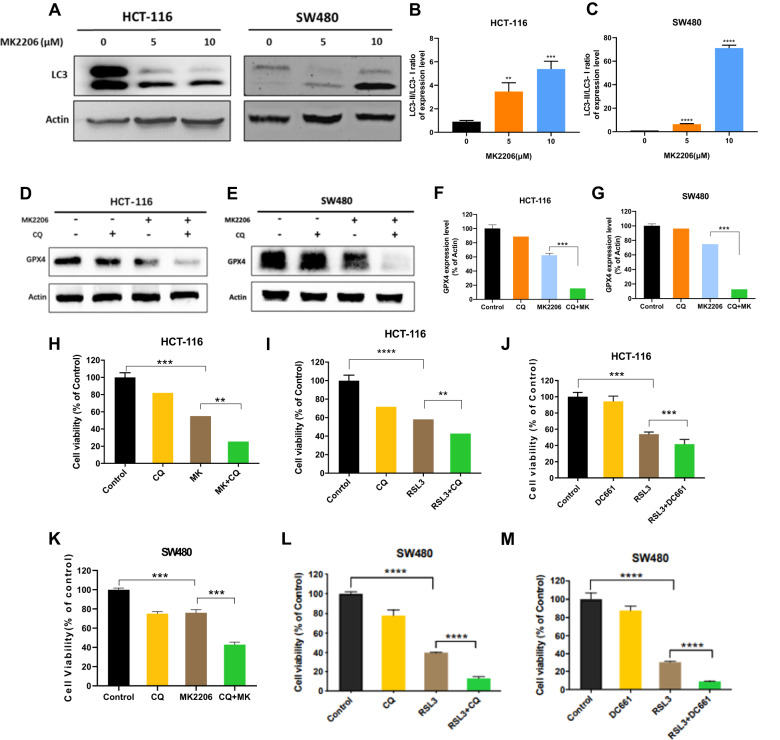
The relationship between MK2206 and autophagy. **A**–**C** The expressions of LC3 and β-Actin were detected after treatment with MK2206. **D**–**G** GPX4 and β-Actin expressions were detected after treatment with MK2206 and CQ. **H**–**M** cell proliferation was detected using the CCK-8 assay after treatment with MK2206, CQ, RSL3 and DC661.

To confirm whether MK2206 caused autophagy while triggering ferroptosis, multiple doses of MK2206 were used to treat CRC cells and LC3 expression levels were checked and measured. As the results shows, the ratio of LC3-II/LC3-I was a concentration dependent phenomenon (Fig. 6A–C), indicating that MK2206 induced autophagy. And it was also confirmed the occurrence of autophagy by cell immunofluorescence analysis after using MK2206 (Fig. S6C). In the further experiments, GPX4 expression levels were significantly reduced following the combined application of MK2206 with CQ compared to MK2206 alone (Fig. 6D–G). Proliferation ability of HCT-116 and SW480 cells were detected by EDU following treatment with MK2206, MK2206 and CQ, RSL3 and RSL3 and CQ (Fig. S6A, B). What’s more, the cell proliferation ability of MK2206 combined with CQ, RSL3 combined with CQ and RSL3 combined with DC661 was further reduced by the CCK8 assay (Fig. 6H–M). Thus, autophagy plays a protective role against AKT-inhibition induced ferroptosis in colorectal cancer, and blocking autophagy can enhance the ferroptosis stimulated by MK2206.

### The combinational therapy of MK2206 and CQ improved the antitumor effect in vivo

**Fig. 7:**
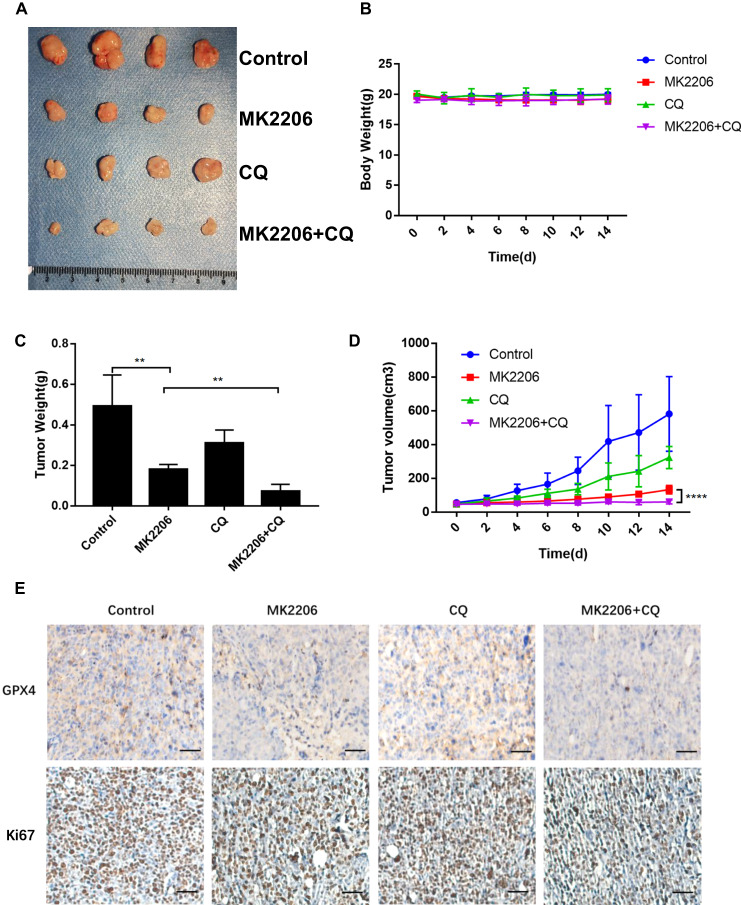
The antitumor effects of MK2206 and CQ in vivo. **A** tumor images of xenograft tumors. **B** The mice body weight was measured twice daily after treatment. **C** The average tumor weight was calculated. **D** The tumor volume was measured twice daily after treatment. **E** IHC staining of GPX4 and Ki-67.

**Fig. 8:**
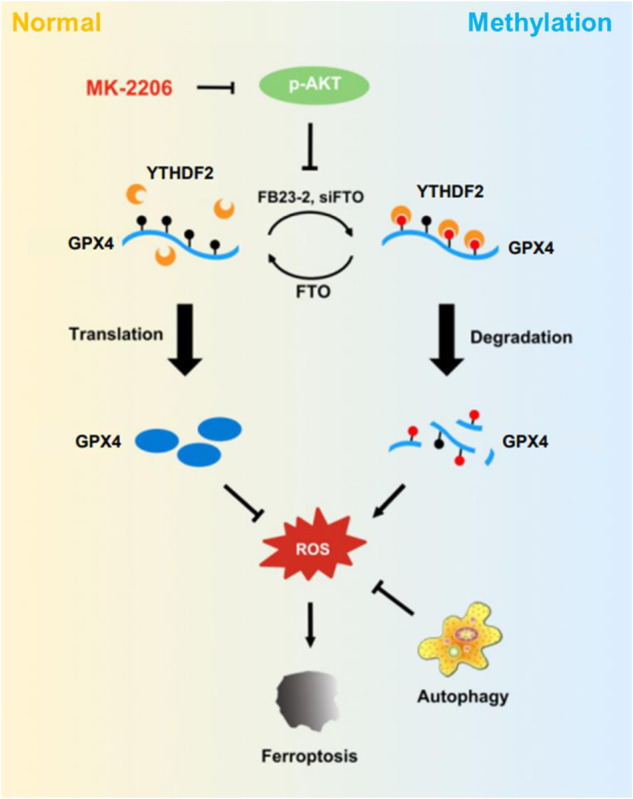
Mechanistic diagram showing the whole process of the research. Targeting AKT leads to ferroptosis through methylation.

To verify the above results in vivo, nude mice were used to construct a xenograft CRC model to see if the combination of MK2206 and CQ could achieve better anti-tumor effects. In the end, the tumor volume and weight of the MK2206 treatment group were lower than the control group, while the tumor volume and weight of the combination with MK2206 and CQ treatment group were lower than the MK2206 group and there were no significant change in mice weight (Fig. 7A–D). The results showed that the combinational therapy possessed a better antitumor effect. Additionally, we stained GPX4 and Ki-67 to detect the expression levels in the tumor tissues by immunohistochemical staining and obtained the corresponding trends (Fig. 7E), which were coincident with the experiments in vitro. Taken together, the above results show that MK2206 can inhibit CRC progression by triggering ferroptosis in vitro and in vivo, and that inhibiting autophagy can strengthen this effect. Finally, a mechanistic diagram of the ferroptosis regulation based on m6A and autophagy is presented (Fig. 8).

## Discussion

The PI3K-AKT signaling pathway plays a significant role in the occurrence and development of multiple tumors [5]. Currently, activation of the PI3K-AKT signaling pathway stimulates the tumor cells proliferation and growth, with P-AKT serving as the main form of AKT [24]. In previous studies, Yi et al. found that the PI3K-AKT-mTOR signaling pathway suppress ferroptosis via SREBP-mediated lipogenesis, and that Inhibiting this pathway could enhance the induction effect of ferroptosis and strengthen the effectiveness of tumor treatment [25]. Cai et al. discovered that inhibiting AKT-mTOR could induce the reduced of GPX4 in glioblastoma. Zhang et al found that mTORC1 promotes GPX4 protein synthesis partly through the mTORC1-4EBP signaling axis [26, 27]. To explain the relationship of 4EBPI and GPX4 after MK2206 treatment, P-4EBP1, 4EBP1 were detected with P-Akt, Akt and GPX4. As a result, P-4EBP1 declined significantly after 1 or 2 uM MK2206 treatment in HCT-116 cells, however, GPX4 did not have a marked decrease in this condition. Furthermore, 5 or 10 uM MK2206 treatment reduced GPX4 evidently but not P-4EBP1. In addition, P-4EBP1 declined inconspicuously after 1, 2, 5 or 10 uM MK2206 treatment in SW480 cells. Meantime, similar trends were observed between P-Akt and GPX4, which indicated that GPX4 is the downstream of Akt but not 4EBP1. Therefore, a regulatory pathway have been discovered that is different from this pathway in colorectal cancer cells. In this study, inhibiting AKT phosphorylation clearly inhibited tumor growth in vivo and in vitro, and the expression levels of FTO and GPX4 were downregulated (Figs. 1, 7). In existing studies, the down-regulation of GPX4 was closely related to the occurrence of ferroptosis [28], and FTO is the key demethylase during the regulation of m6A methylation modification [29]. Therefore, it inspired us to explore the relationship between methylation modification and ferroptosis in CRC due to the simultaneous decrease in the expression levels of FTO and GPX4 following AKT inhibition.

Ferroptosis regulates cell death by iron accumulation and lipid peroxidation, which is significantly different from other kinds of cell death and plays pivotal roles in a great many biological processes [30]. Additionally, ferroptosis also broadly regulates the cancer process. In gastric cancer, it was shown that ELOVL5 and FADS1 enhance the sensitivity to ferroptosis, increasing anti-tumor effect [31]. In breast cancer, inhibition of SLC7A11 could induce ferroptosis [32]. Further recognition of ferroptosis in the CRC will bring new perspectives for tumor treatment. In 2014, Wan et al. reported that GPX4 is a key regulator of ferroptosis [12], which could protect tumor cells from ferroptosis thus affecting tumor therapy [33, 34]. Therefore, exploring effective GPX4 regulation mechanisms is critical for tumor therapy based on ferroptosis. At present, it is reported that AKT and ferroptosis have a regulatory relationship [25, 35]. However, the detailed mechanism by which AKT and GPX4 interact remains unclear. In our study, we used the AKT inhibitor, MK2206, to treat CRC cells and found that ferroptosis occurred after treatment of MK2206 and GPX4 was obviously inhibited in this process, which enhanced ROS production. Attractively, the expression level of the demethylase of m6A modification, FTO, was also found to decrease in the process. Therefore, the underlying mechanisms between methylation and AKT inhibition-induced ferroptosis were further explored.

As the most frequent form of internal RNA modification in eukaryotes, m6A methylation plays a variety of roles in the normal and pathological processes of the body. In the majority of cases, m6A plays a carcinogenic role in the cancer tumorigenesis and progression [35–38]. The methylation of m6A is an important regulatory mechanism in CRC. Li et al. found that METTL3 could promote tumor progression via IGFBP2 in CRC [39]. Meantime, Chen et al. found that METTL14 could inhibit tumor progression though miR-375/YAP1 pathway in CRC [40]. What’s more, recent research has proclaimed the relevance between m6A and ferroptosis. Fan et al. found that METTL14 triggered SCL7A11 though YTHDF2 to block ferroptosis in hepatocellular carcinoma [41]. In addition, YTHDC2 could act as an endogenous ferroptosis inducer after targeting SCL3A2 in lung adenocarcinoma [42]. However, the mechanism especially the relationship between m6A and GPX4 remains unclear. Here, we firstly reported that m6A methylation could regulate ferroptosis via increasing the m6A methylation level by inhibiting FTO, thus promoting its degradation mediated by YTHDF2. Furthermore, we predicted the potential methylation modification sites on GPX4 mRNA by SRAMP [43], and confirmed the methylation site 193 on GPX4 mRNA was regulated by FTO. In the process of methylation regulation, m6A readers can identify the m6A modification sites and have a great responsibility for the outcomes of m6A-modified mRNAs [44], of which YTHDF2 could recognize m6A methylation modification sites and promote mRNA degradation to regulate cell progression [23]. Our study also found that YTHDF2 was the key m6A reader for the methylation modification of GPX4 and played a role in the degradation of GPX4.

In addition to the regulation of methylation, there are many regulatory mechanisms that influence ferroptosis [45]. For example, previous studies have indicated that autophagy plays an essential role in regulating the occurrence of ferroptosis [46, 47] and ferritinophagy-mediated ferroptosis could be induced by nanoparticles [48]. It has been reported that autophagy can be either cell death protective or inhibitory in cancer [49]. In our previous study, we found that autophagy possessed a protective function in CRC [22, 50]. Interestingly, in this work, it was found that the combinational treatment of MK2206 and CQ could significantly reduce the expression level of GPX4 and promote the occurrence of ferroptosis. Importantly, both cell and animal studies confirmed that the inhibition of autophagy could strengthen the anti-tumor effect of MK2206 (Figs. 6, 7, S2), indicating that autophagy might play cell death protective effect in CRC after inhibiting AKT. These provided a potential therapeutic target for tumor resistance and new strategies for the combination therapy in CRC.

Collectively, our study demonstrated a novel underlyingly ferroptosis regulation mechanism, which targeting AKT stimulated colorectal cancer ferroptosis through regulating demethylation of FTO induced GPX4 degradation in a YTHDF2-dependent manner and the accurate m6A methylation modification site 193 was confirmed. At the same time, the cell death protective role of autophagy in the induction of ferroptosis through AKT inhibition was identified. These finding may furnish some valuable guidance for the treatment approaches of CRC.

## Materials and methods

### Cell culture

HCT-116 cell lines and SW480 cell lines were purchased from American Type Culture Collection. HCT-116 cell lines were cultured in DMEM medium (Gibco, Carlsbad, USA) and SW480 cell lines were cultured in L-15 medium (KeyGEN BioTECH, China) containing 10% fetal bovine serum (FBS; Gibco, USA), 1% penicillin and streptomycin (NCM Biotech, China) at 37 °C with 5 % CO_2_. All cell lines used in the study are free of mycoplasma contamination.

### Reagents and antibodies

The Chloroquine (CQ) was obtained from solarbio (Beijing, China). RSL3, FER-1, FB23-2, DC661, Z-VAD and NAC were purchased from Selleck Chemicals (Houston, TX, USA). MK2206 was obtained from Topscience (Shanghai, China). Primary antibodies against m6A, LC3, Ki67, AKT, p-AKT, FTO, YTHDF1, YTHDF2, YTHDF3, FN1 and β- actin were obtained from Proteintech (Wuhan, China); GPX-4, SIRT6 and p-SIRT6 were obtained from Abcam (Cambridge, UK).

### Western blotting

Protein samples were prepared by using cell lysis buffer for Western and IP (Beyotime, China) and SDS-PAGE gels were used. PVDF membranes (Millipore, USA) were used for transfer and blocked with 5% BSA for 1 h at 25 °C. Incubating the corresponding primary antibody at 4 °C overnight, and then incubating the secondary antibody was at 25 °C for 1 h. The relative expression level of protein was calculated using Chemiluminescence imaging system. All western blots are in the Supplemental Material.

### RT-qPCR

Total RNA was extracted and reversed into cDNA using TRIZOL reagent and HiScript Q RT SuperMix for qPCR Kit (Vazyme, Nanjing, China). The primer sequence used in the experiment is shown in Supplementary Table S1. Then using ChamQ Universal SYBR qPCR Master Mix Kit (Vazyme, Nanjing, China) to measure relative mRNA expression levels and 2- ΔΔ Ct is used to count the expression level relative to β-actin.

### MeRIP-qPCR

Using MeRIP Kit (Epigentek, USA) to obtain mRNA fragments from Total RNA that had m6A modification site, then we used specific primer sequences to detect GPX-4 methylation modification levels by qPCR.

### Immunohistochemistry staining

Tumor tissue was fixed with 4% paraformaldehyde for 24 h for immunohistochemistry (IHC). After dehydration of alcohol and xylene and paraffin embedded tissue immediately, the tumors were cut into slices into 5 μm. In short, 1% hydrogen peroxide was used as a blocker to block antigen retrieve. Incubating the corresponding primary antibody at 4 °C overnight, subsequently incubating the secondary antibody at room temperature for 30 min. After using DAB chromogenic agent, dehydrate the tissue, and use neutral gum chip. The results were obtained under the optical microscope by three experienced researchers [51].

### m6A dot blot assay

Total RNA, obtained from cells and tissues, were denatured at 70 °C within 10 min, samples were placed on ice for no less than 2 min immediately and divided into subgroups of 200 ng, 400 ng and 800 ng. The equal volume of RNA samples was loaded to a nylon membrane (Beyotime, Shanghai, China). Then put the nylon membrane in an 80 °C drying oven for 2 h to bind the RNA to the nylon membrane. Nylon membrane block and antibody incubation refer to Western blotting. The results were also detected as Western blotting.

### Cell viability assays and colony formation

Inoculate 5 × 10^3^ CRC cells per well in 96-well plate. After incubation for 24 h, replaced the normal medium with different treatment medium. Finally, we measured absorbance (OD450) at different time points by 96-well plate reader. Cell Counting Kit-8 (CCK-8) was purchased from APExBIO (Houston, USA). For colony formation, Inoculate 1 × 10^3^ CRC cells per well into 6-well plate. After incubating 2 weeks and after relevant treatment, fixation with polyformaldehyde and proper cleaning, staining with crystal violet solution, and finally scan and image

### ROS detection and measurement

The occurrence of ROS was measured by ROS Assay kit (Beyotime, Shanghai, China). After 24 h of different treatment, mixed 10 μM/ML DCFH-DA in medium, and then incubated together for 30 min. After incubation, PBS was used to wash it three times, and then used fluorescence microscope to obtain images. The ROS-positive cells were calculated by Image J as result quantification.

### Cell transfection and RNA interference

The shRNA plasmid of AKT was purchased from Hansheng Technology. All siRNAs were purchased from genepharma (Shanghai, China). 293 T cells were used to construct the shAKT stable cell line. The plasmid was transfected into 293 T cells by Lifefectamine 2000 (Invitrogen, USA), and the packaged plasmid was assembled and transfected into HCT-116 cells at 24 h, 48 h and 72 h respectively. The siRNAs were imported into target cells by Lipofectamine 2000. After transfected 24 h, we replaced normal medium and continued to incubate cells for 24 h to next treatment.

### EDU assay

Using the EDU reagent to inquire about the cell proliferation ability. The density of CRC cells is 5 × 10^4^ cells were inoculated on 24-well plate. After treated 24 h, they were incubated with 10 μM EDU buffer in the CO_2_ incubator for 2 h, subsequently fixed with 4% formaldehyde for 15 min, washed with PBS, then permeated with 0.1% Triton X-100 for 10 min. EdU solution was added into wells and then used Hoechst to staining of nuclei. Finally, the images were detected by a fluorescence microscope.

### Animal models

The 6–8 weeks old Balb/c female nude mice were divided into 4 groups (Control, MK2206, CQ, MK2206 + CQ) randomly(4/group) and 1 × 106 cells were resuspended at 100 μl PBS were injected subcutaneously to the left armpit of the mice under sterile operation. When the tumor size reached 50 mm^3^, we started treating mice with 90 mg/kg MK2206 [52] by gavage and 25 mg/kg CQ [22] by intraperitoneal injection. Untreated mice were injected with the same volume of 0.9% NaCl and the same volume of sodium carboxymethyl cellulose solution by oral gavage. During the experiment, mouse weight and tumor size were determined once every 2 days and were euthanized after 14 days of different treatments. Tumor volume calculation formula: 1/2*(length × width^2^).

### Statistical analysis

The experiment datas were conducted with GraphPad Prism 7.0 software and were duplicated no less than three times independently. The dates were expressed as the mean + SEM. *P*-values were estimated by using the student’s paired *t*-test.

### Supplementary information

supplementary material file

CDDISCOVERY-23-0697R1 Initial Quality Check

SUPPLEMENTAL MATERIAL Table S1

Fig S1

Fig S2

Fig S3

Fig S4

Fig S5

Fig S6

Initial western blotting

## Data Availability

The data sets used and/or collected in this study were obtained from the corresponding author.
