# Supplementary material for: Targeting AKT induced Ferroptosis through FTO/YTHDF2-dependent GPX4 m6A methylation up-regulating and degradating in colorectal cancer
Source: Cell Death Discov. 2023 Dec 15;9:457. doi: 10.1038/s41420-023-01746-x (PMC10724184; doi:10.1038/s41420-023-01746-x)
Supplement: Supplementary file 1 — supplementary material file [file 41420_2023_1746_MOESM1_ESM.docx]

**
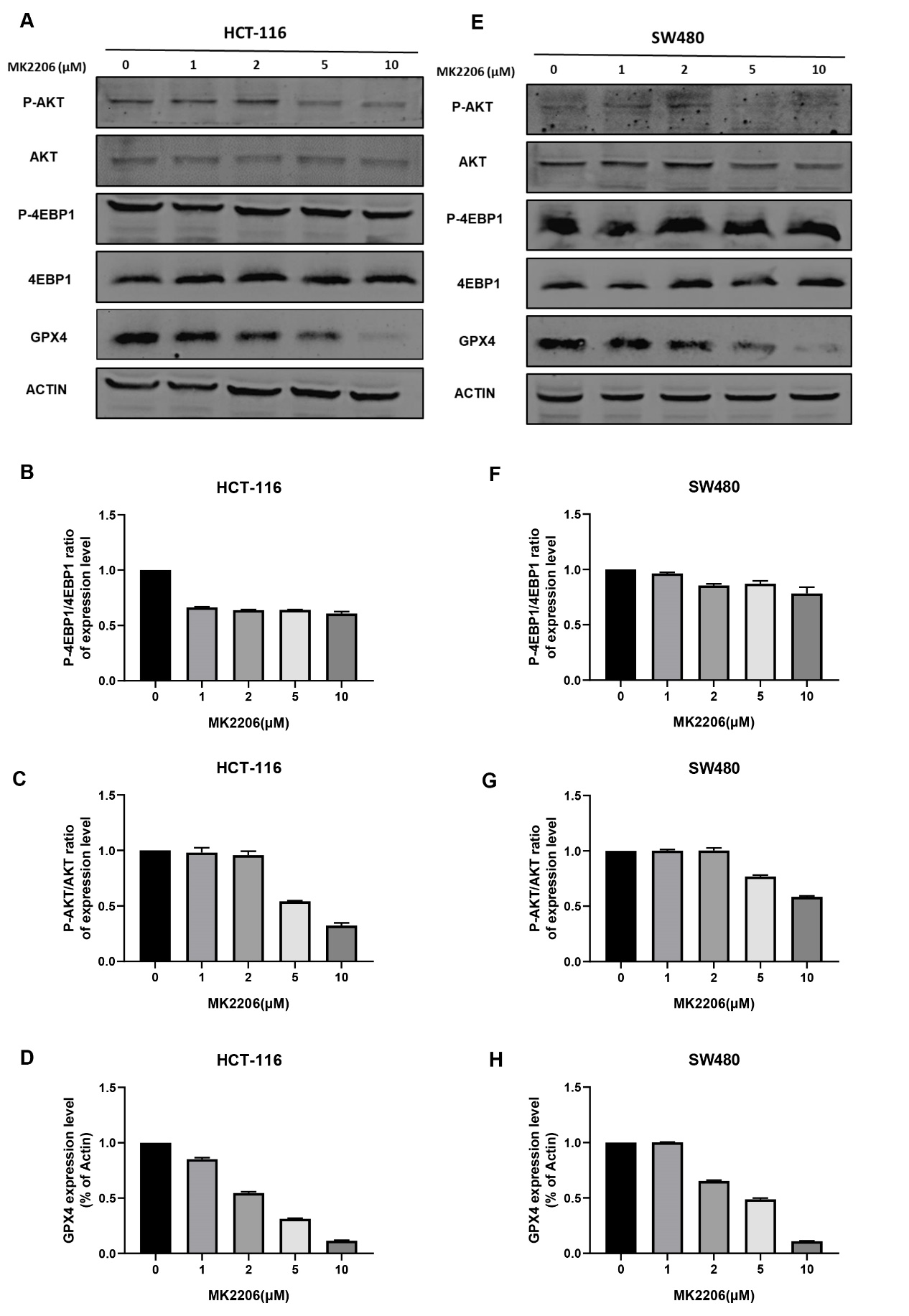
Fig S1：MK2206 inhibited cell proliferation in colorectal cancer. A-H** Effective action concentration of MK2206 was detected and counted in CRC cells.

**
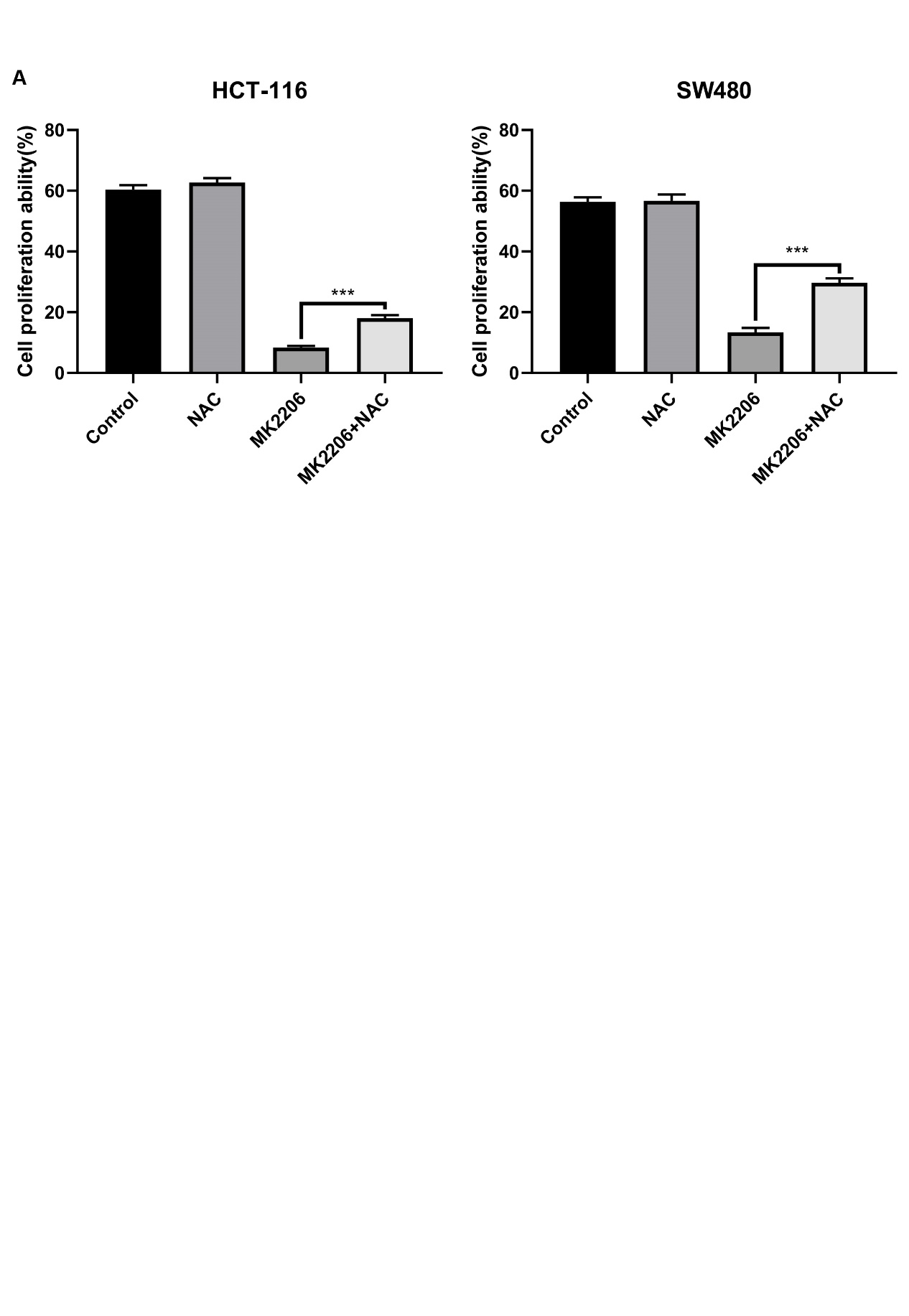
Fig S2：A, B** The EDU assay was counted.**
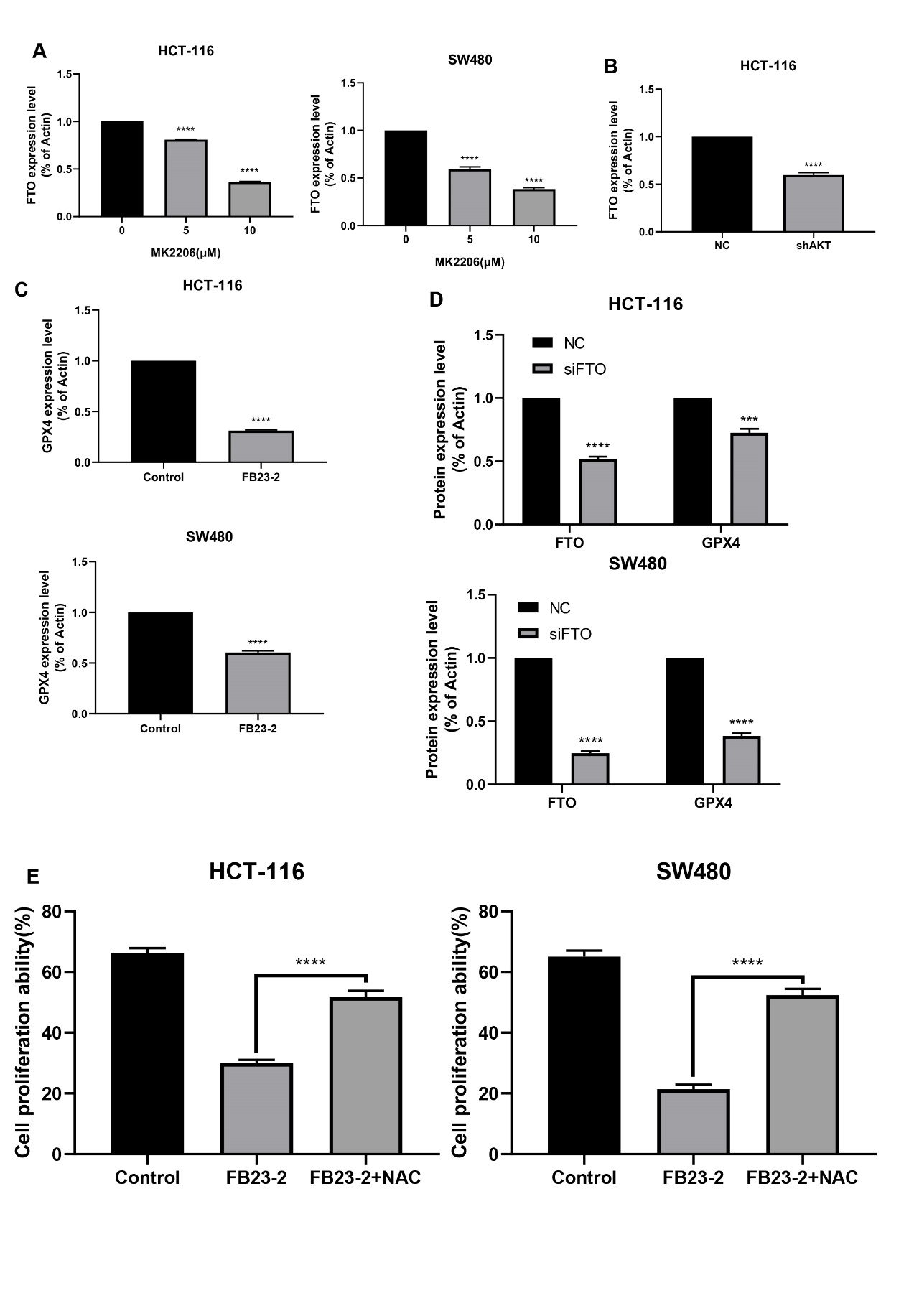
Fig S3: A-D** The results of western blotting were counted. **E** The EDU assay was counted.

**
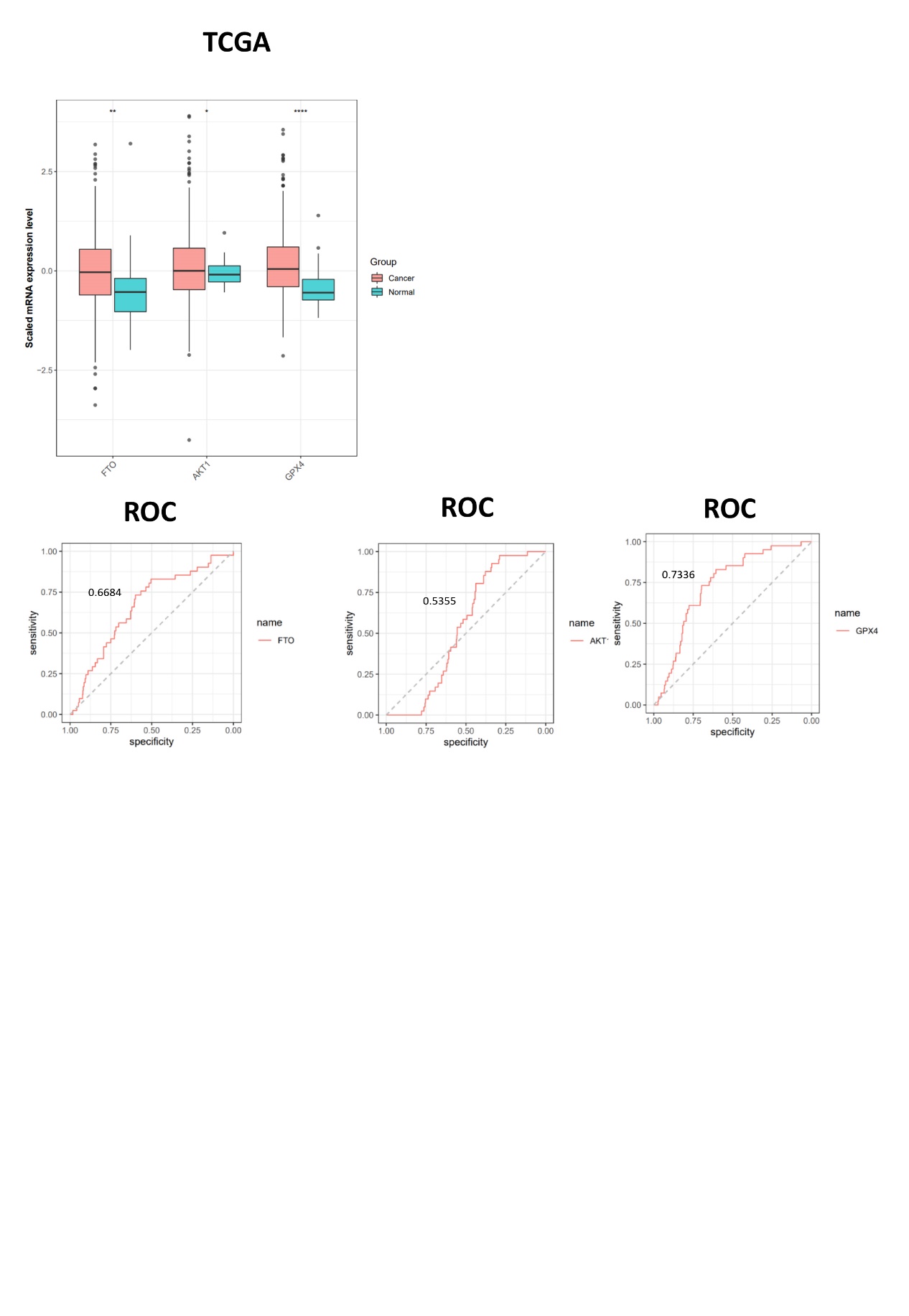
Fig S4：AKT, FTO and GPX4 high expression in colorectal cancer.** The expression of AKT, FTO and GPX4 was high by TCGA analysis.

**
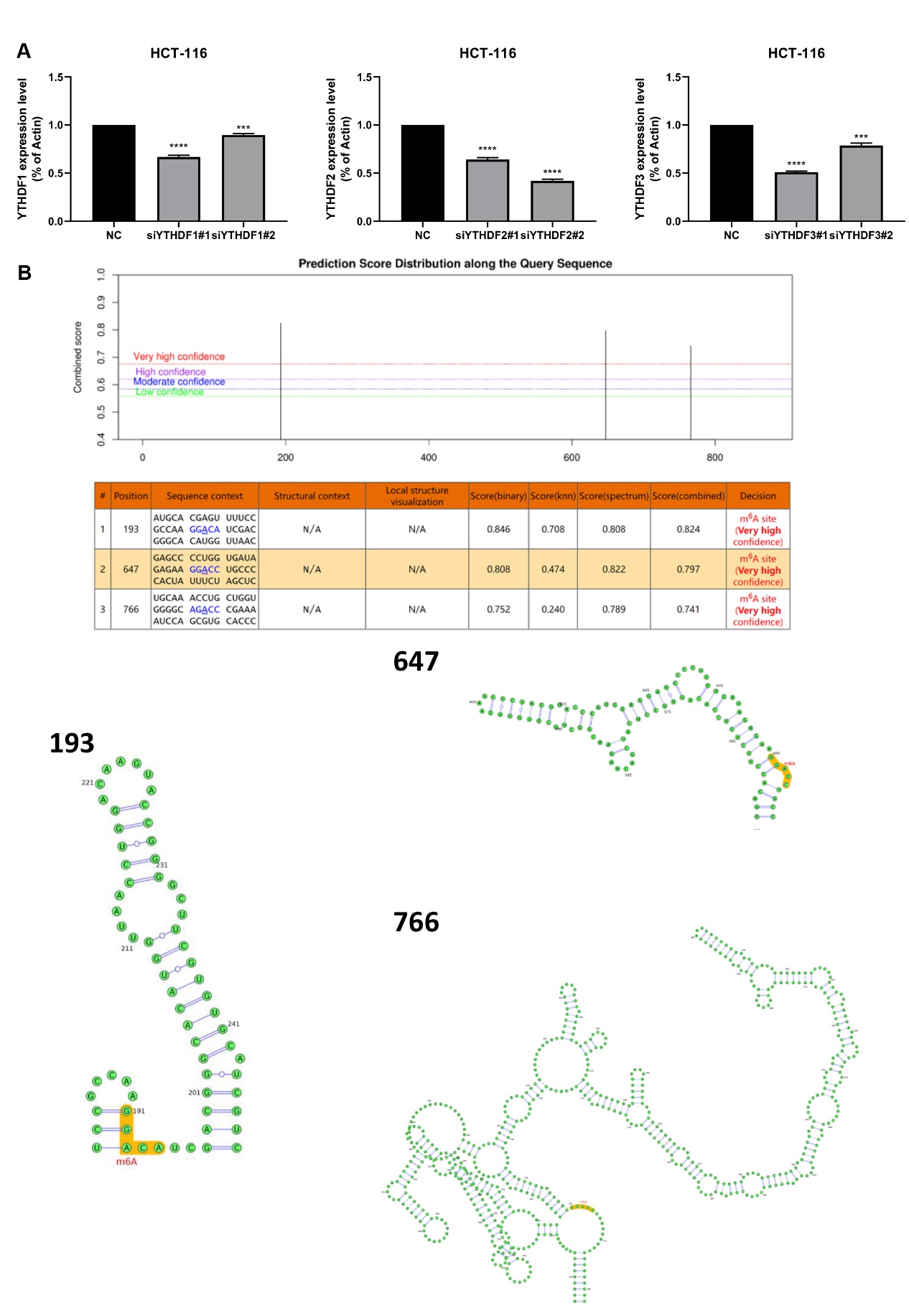
Fig S5：m6A modification sites were predicted by SRAMP. A T**he results of western blotting were counted. B The modification sites 193, 647 and 766 were predicted by SRAMP.

**
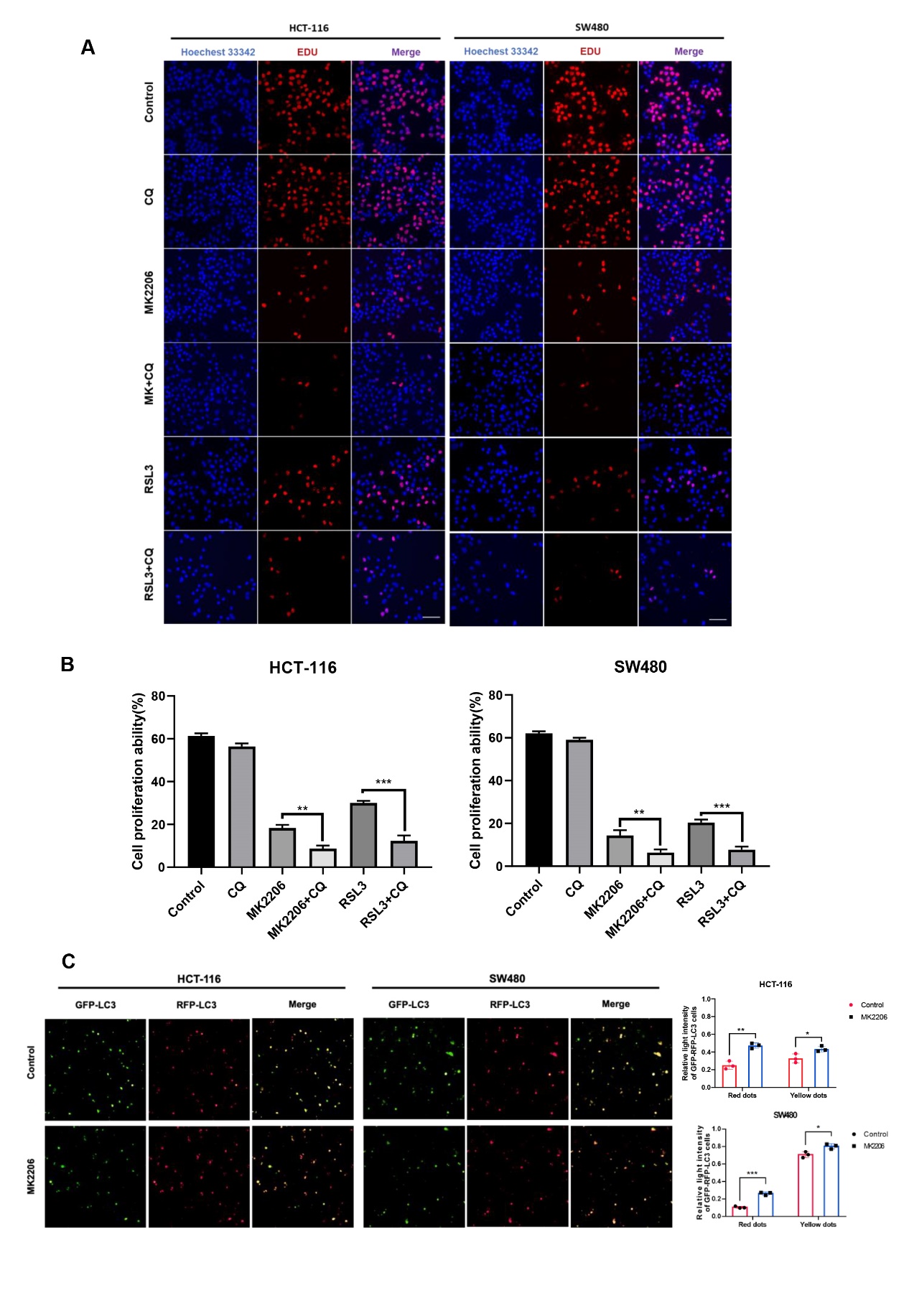
Fig S6：The relationship between autophagy and cell death. A, B** The EDU assay was applied to compare the cell proliferation ability in HCT-116 and SW480 that were treated with MK2206, CQ MK2206+CQ, RSL3 and RSL3+CQ [scale bar, 100μm]. **C** HCT-116 and SW480 stably expressing GFP-RFP-LC3 fusion protein were treated with MK2206 and the results were observed with a confocal microscope (2000 times magnification).

**Table S1: Primer sequence.**

**FTO**

FP: GCTGCTTATTTCGGGACCTG

RP: AGCCTGGATTACCAATGAGGA

**GPX4**

FP: GGGACCATGTGCGCGT

RP: ACTTCGGTCTTGCCTCACTG

**GPX4-1**

FP: CGCCGCGATGAGCCT

RP: GTGACGATGCACACGAAGC

**GPX4-2**

FP: CACCGTCTCTCCACAGTTCC

RP: ACGCTGGATTTTCGGGTCTG

**GPX4-3**

FP: GGACCTGCCCCACTATTTCTA

RP: TTTATTCCCACAAGGTAGCCAG
