# Supplementary figures and images for: Targeting AKT induced Ferroptosis through FTO/YTHDF2-dependent GPX4 m6A methylation up-regulating and degradating in colorectal cancer
Source: Cell Death Discov. 2023 Dec 15;9:457. doi: 10.1038/s41420-023-01746-x (PMC10724184; doi:10.1038/s41420-023-01746-x)

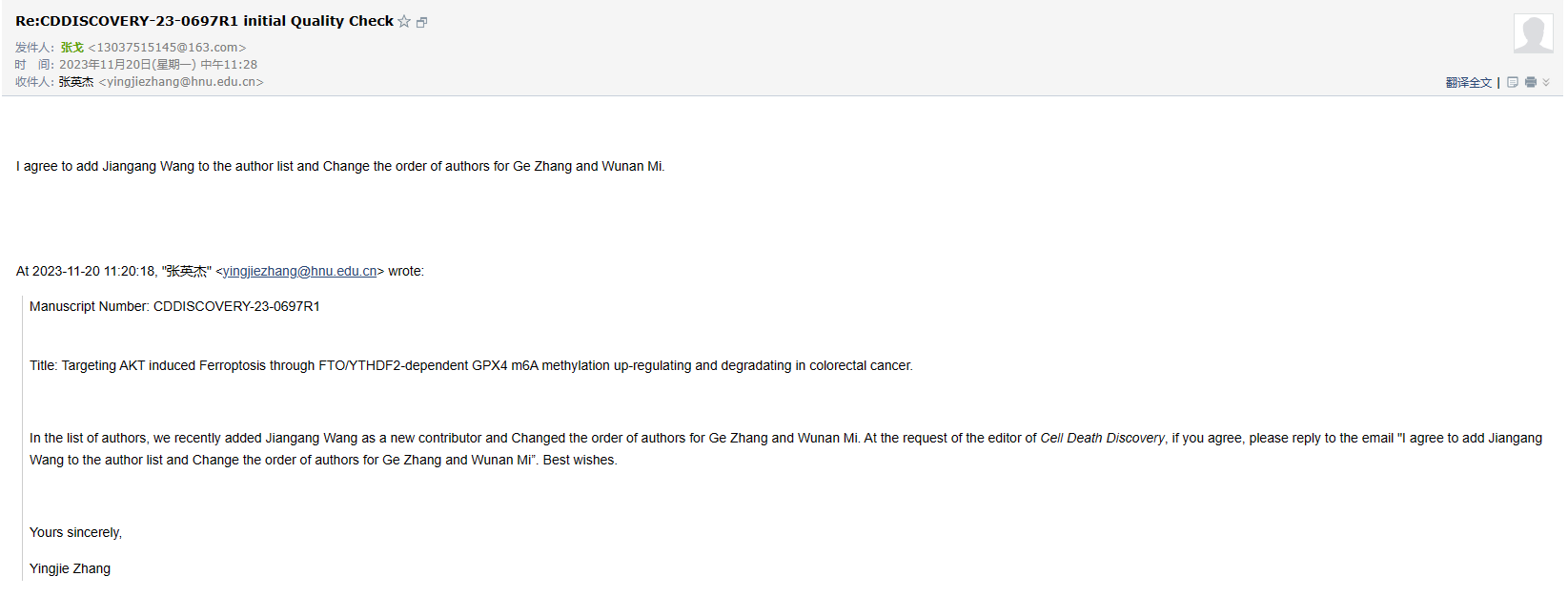


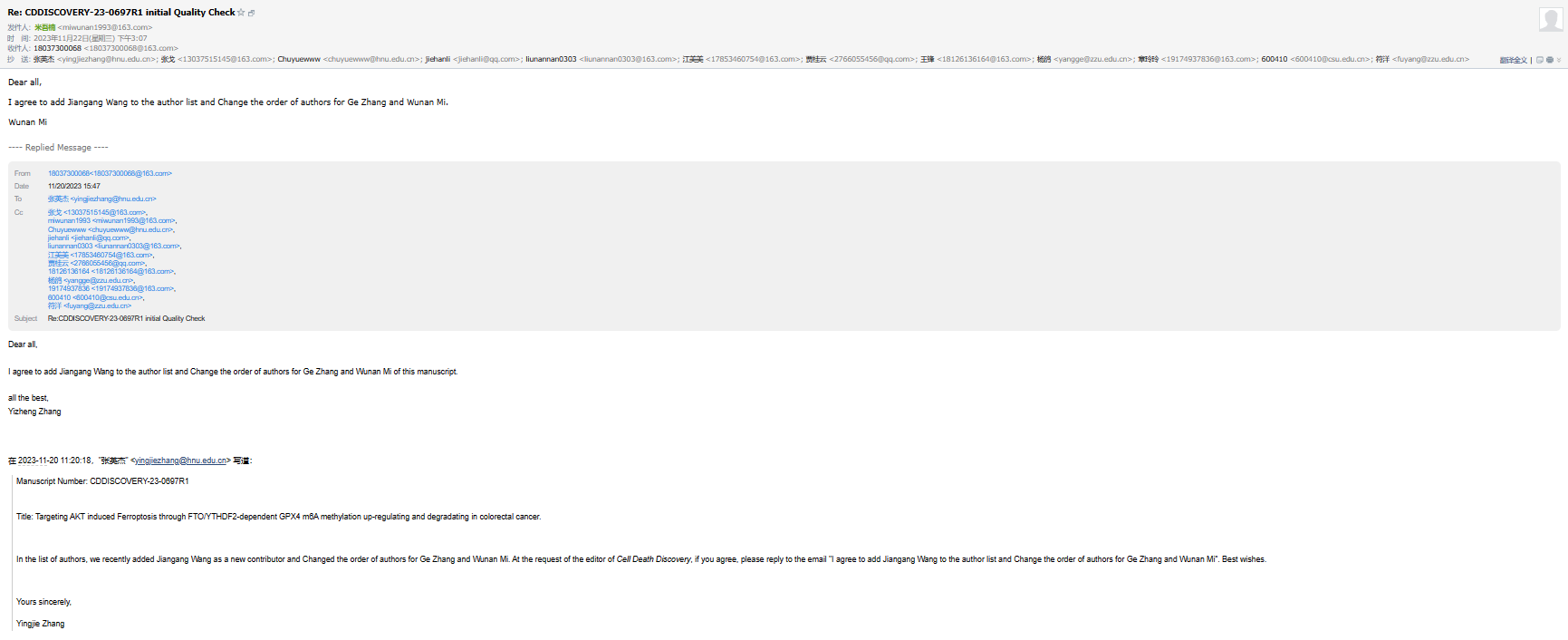


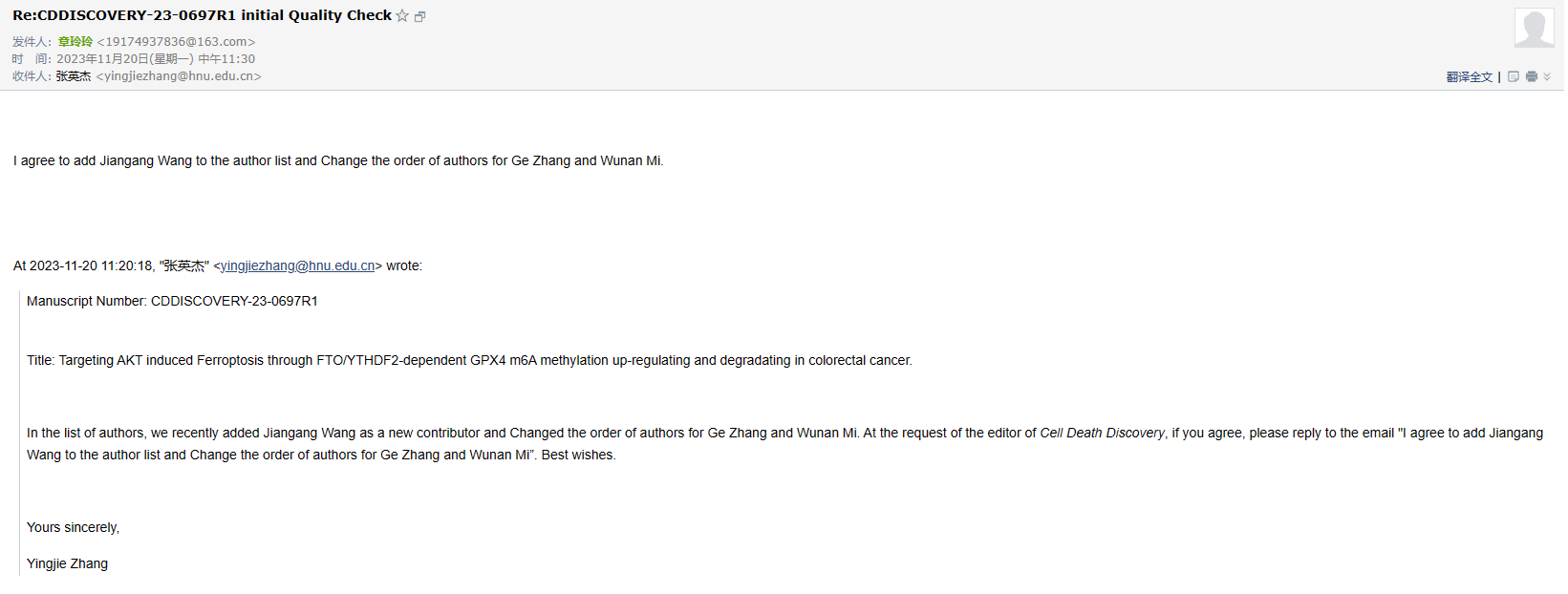


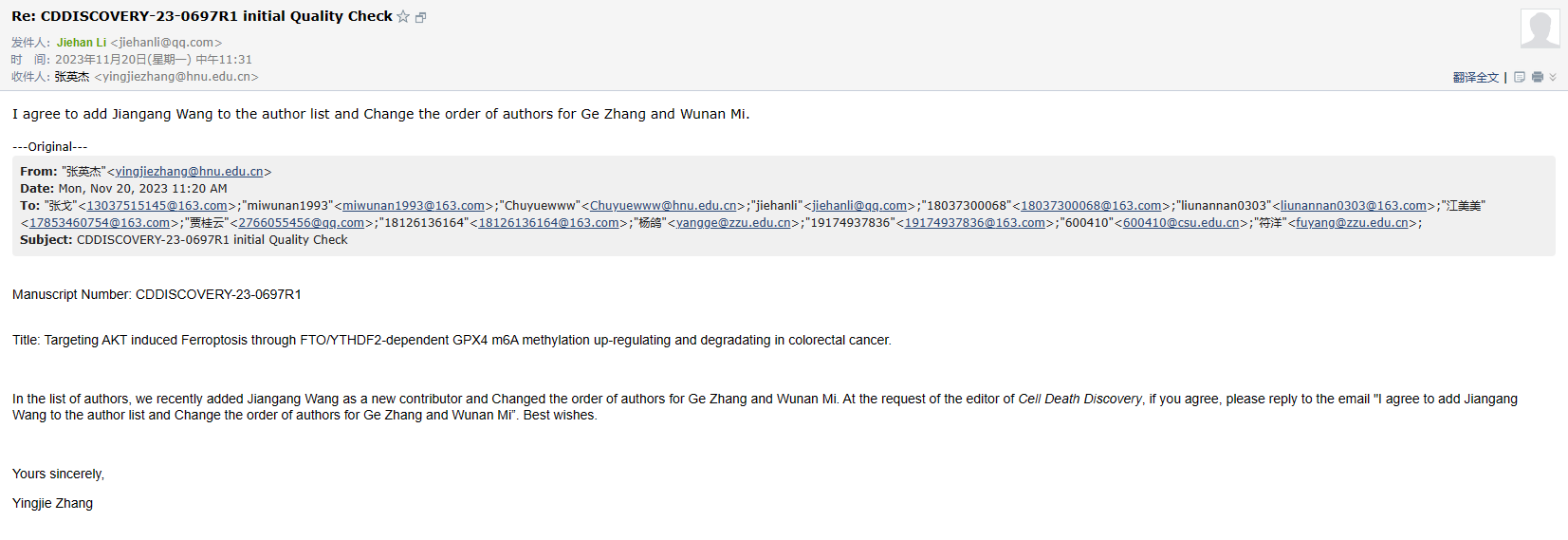


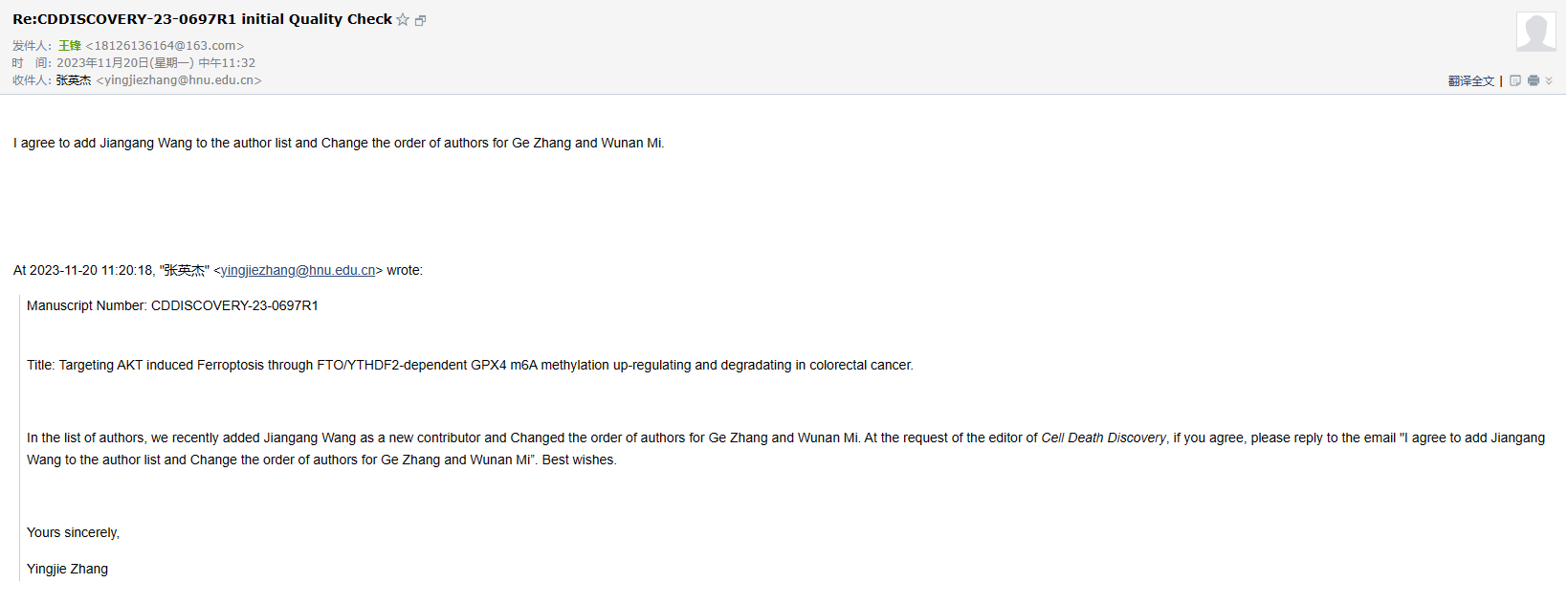


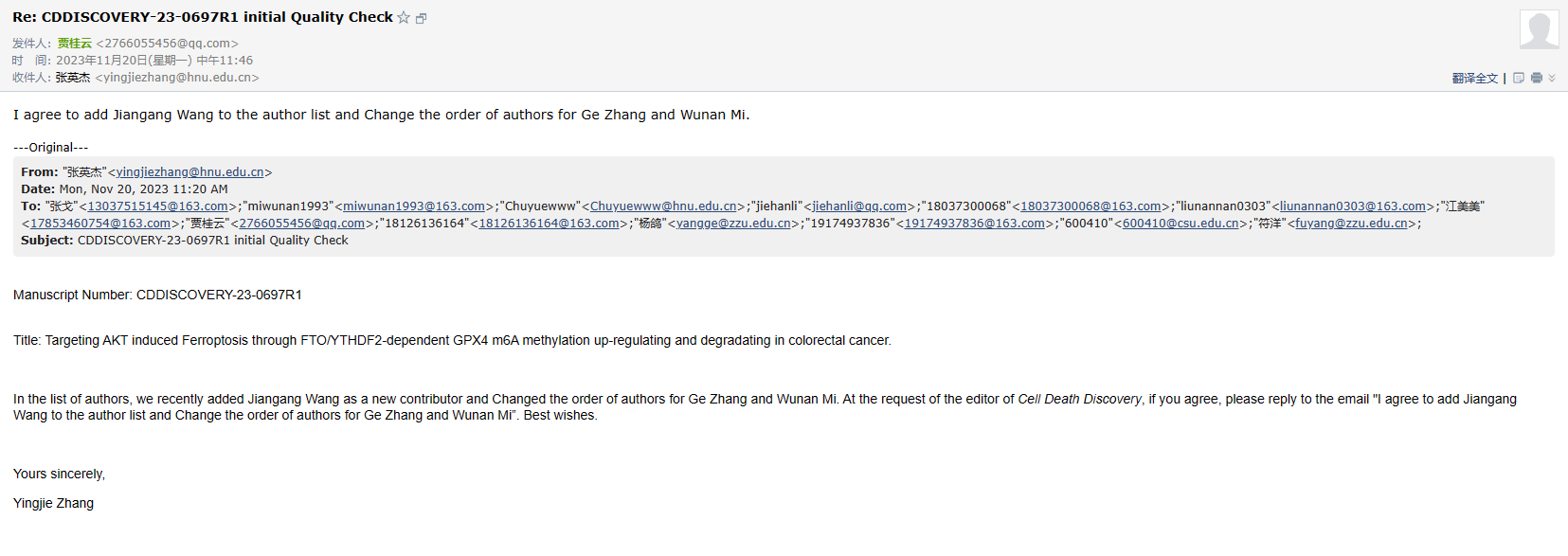


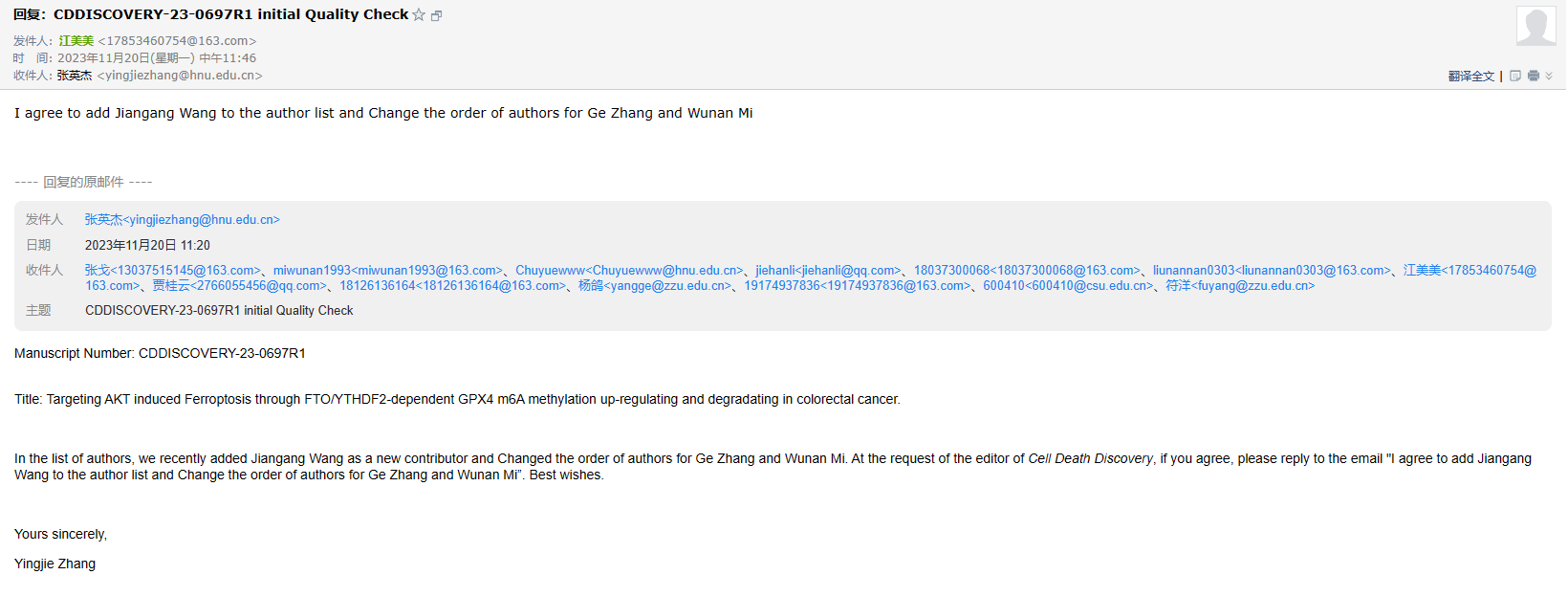


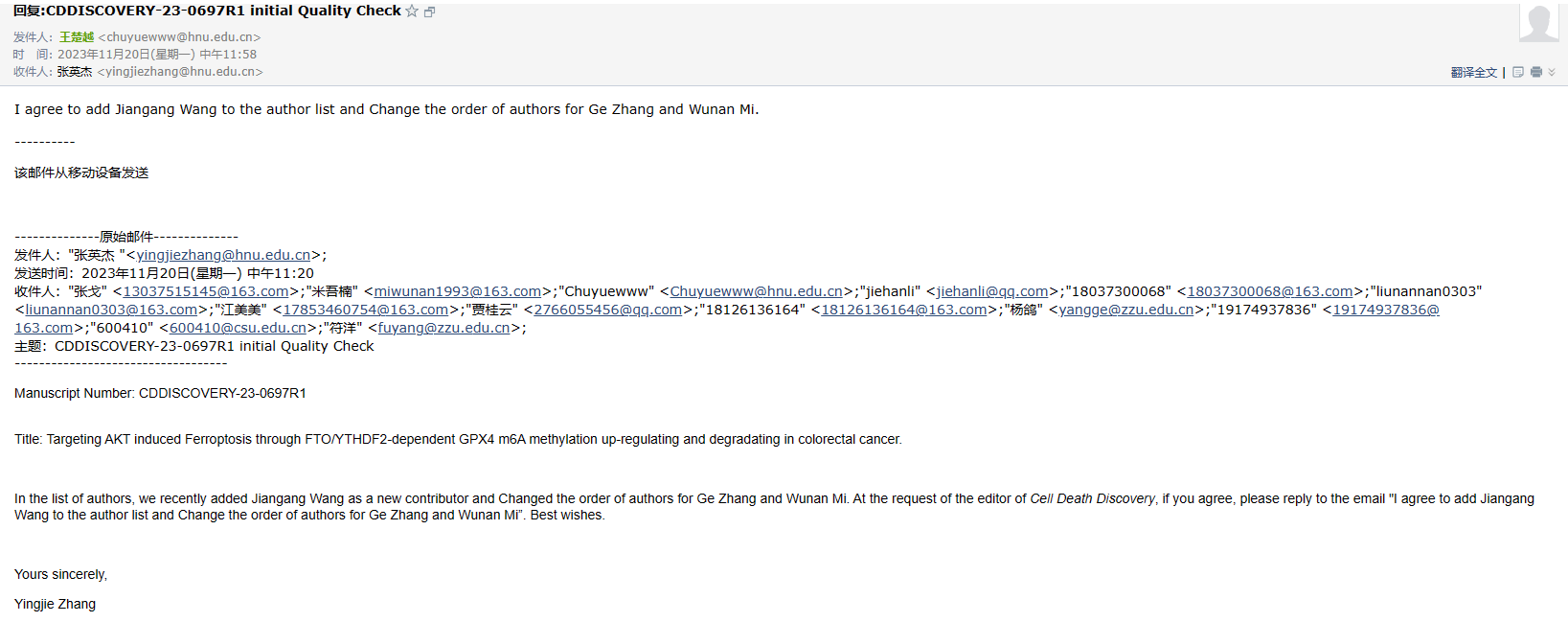


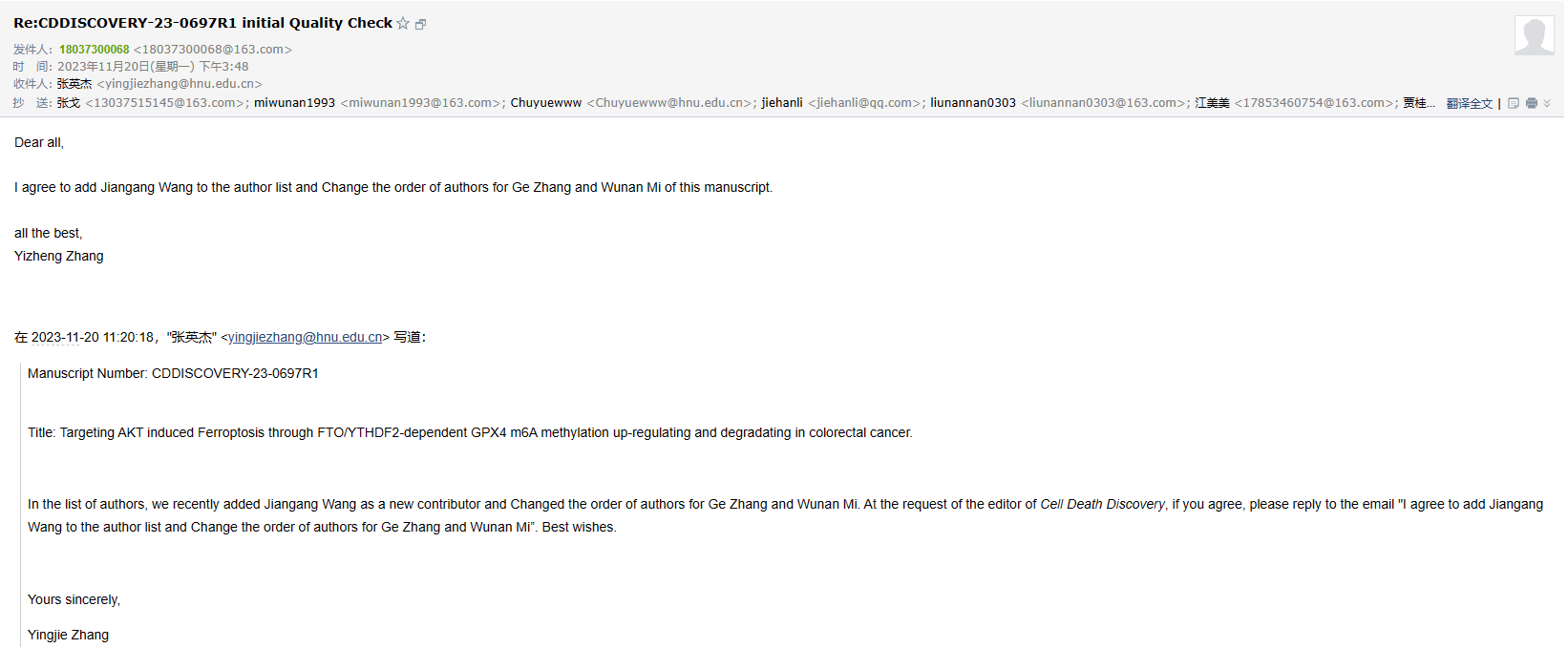


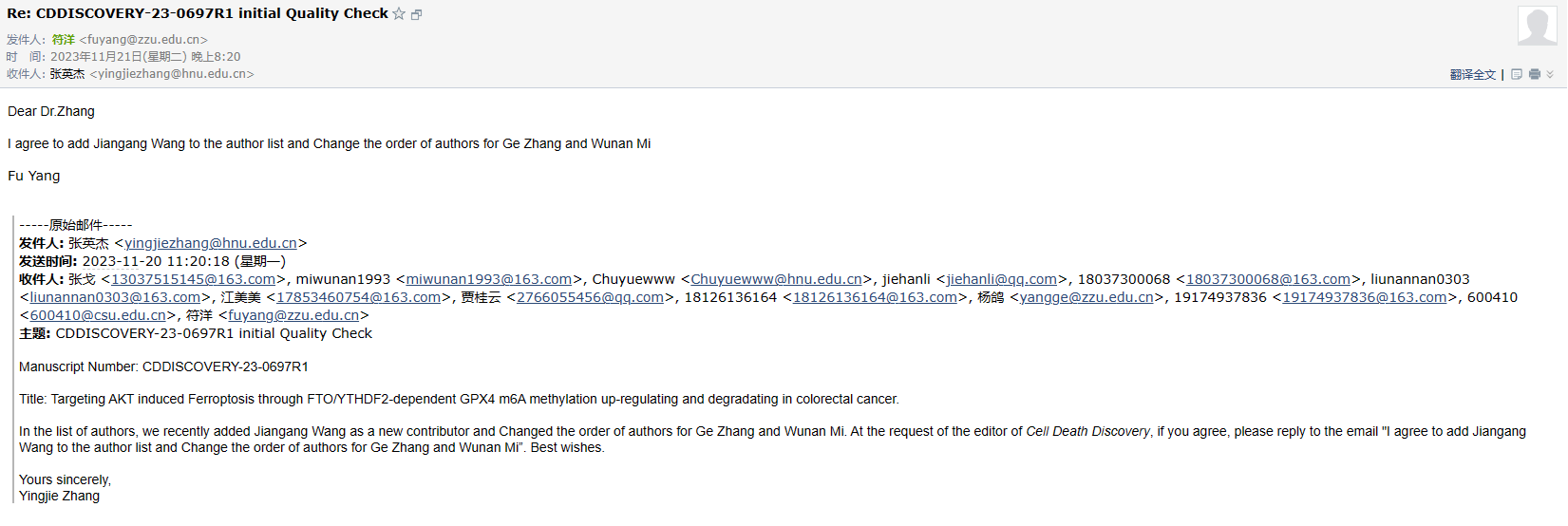


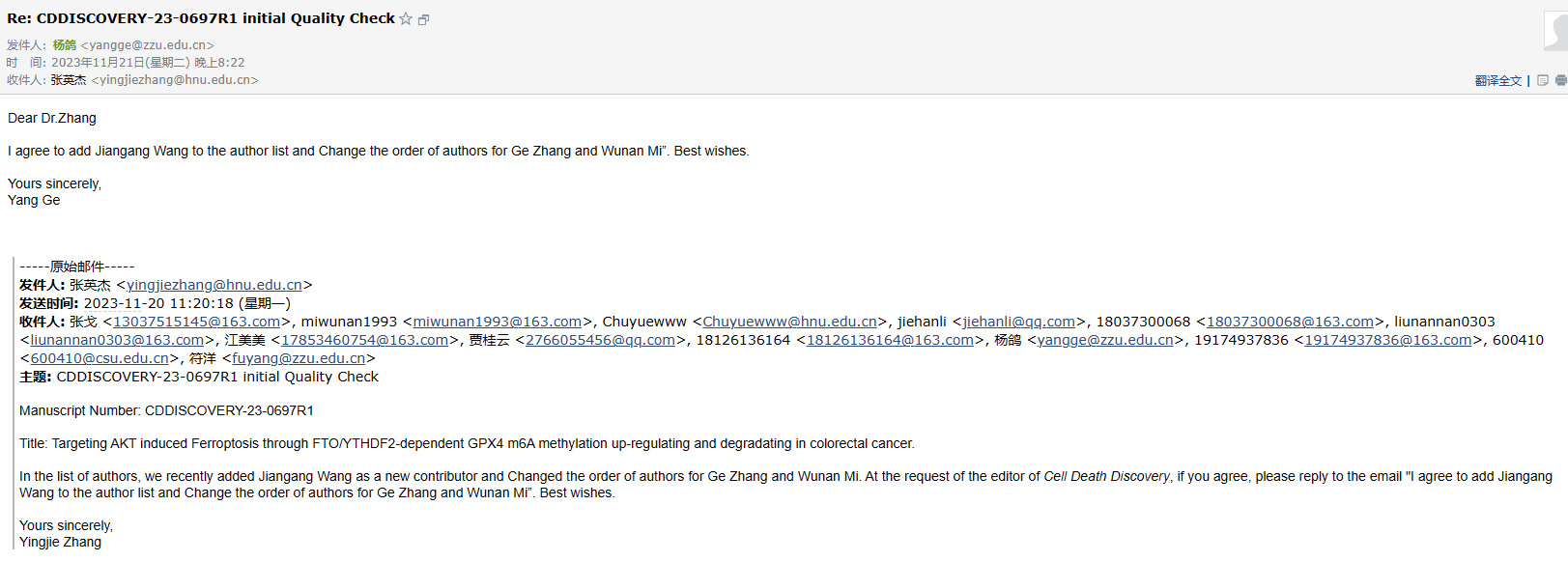


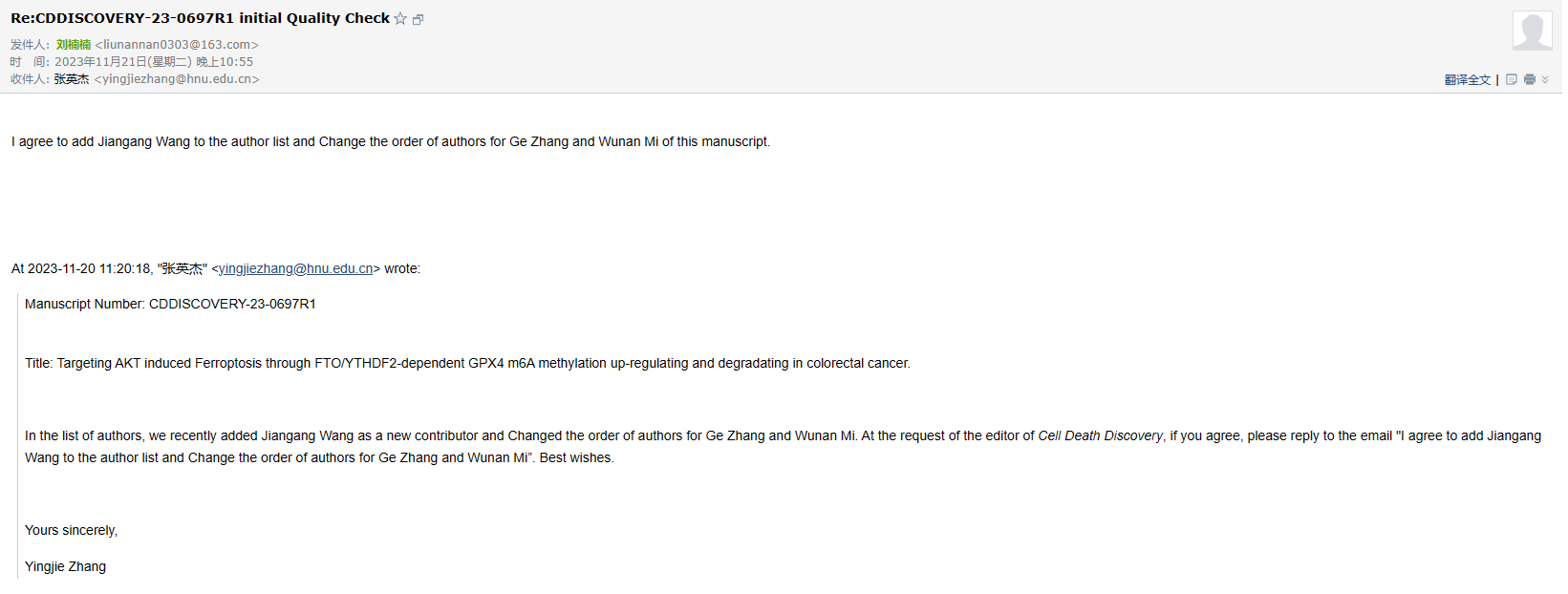


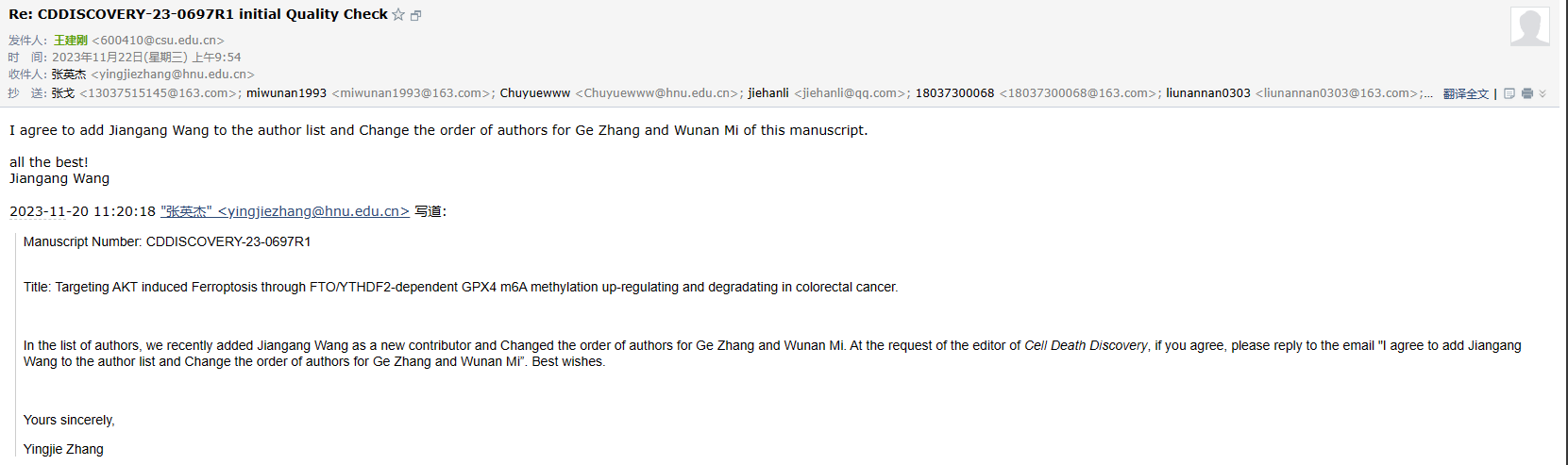

Supplement: Supplementary file 2 — CDDISCOVERY-23-0697R1 Initial Quality Check [file 41420_2023_1746_MOESM2_ESM.docx]

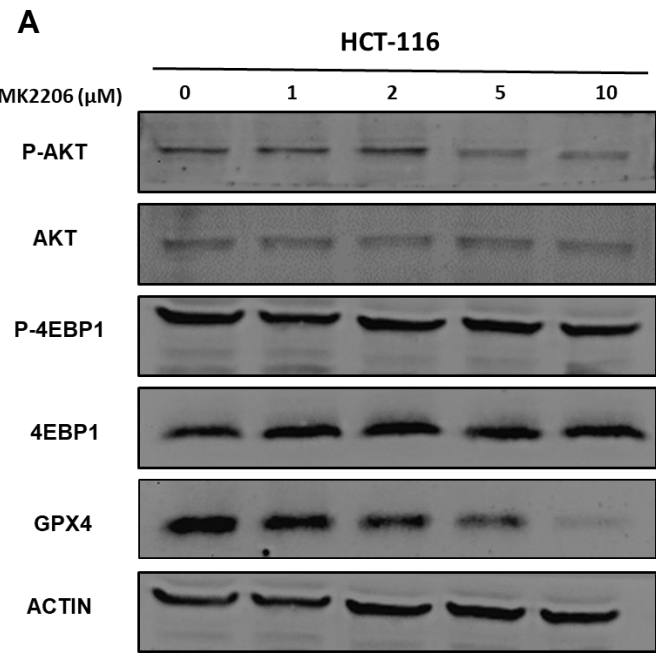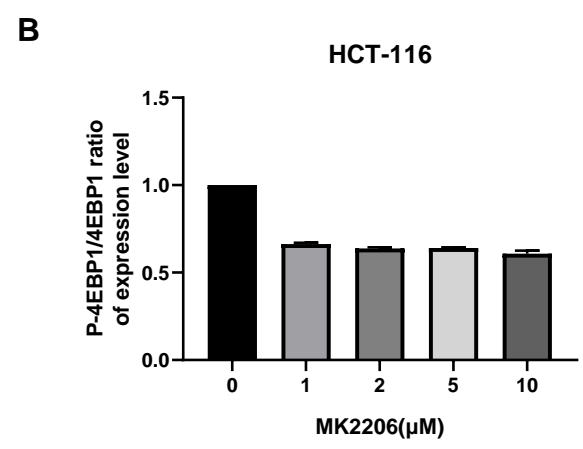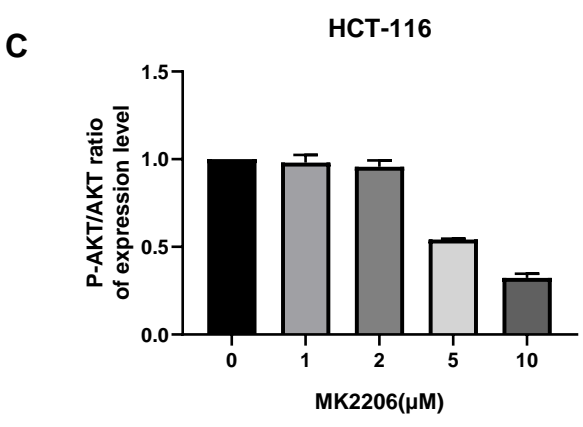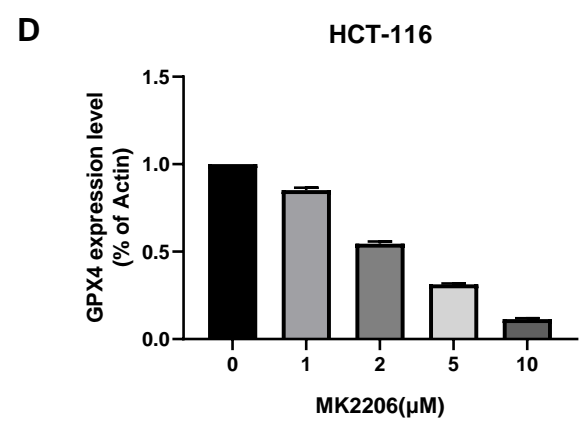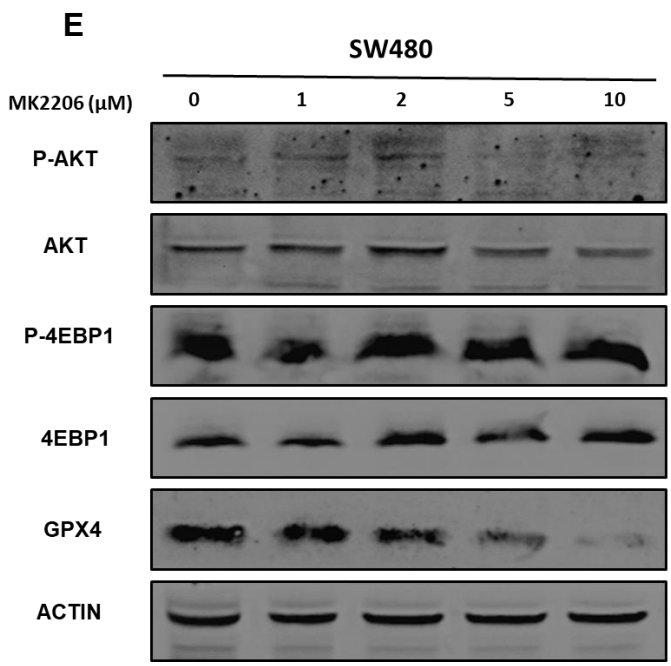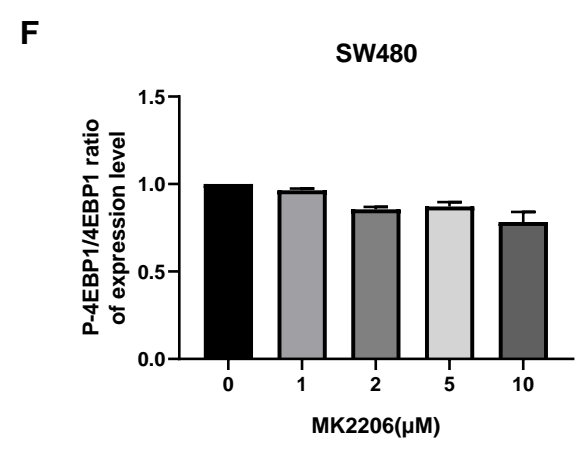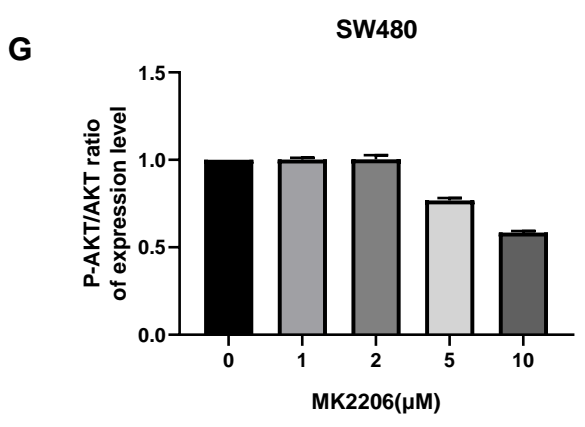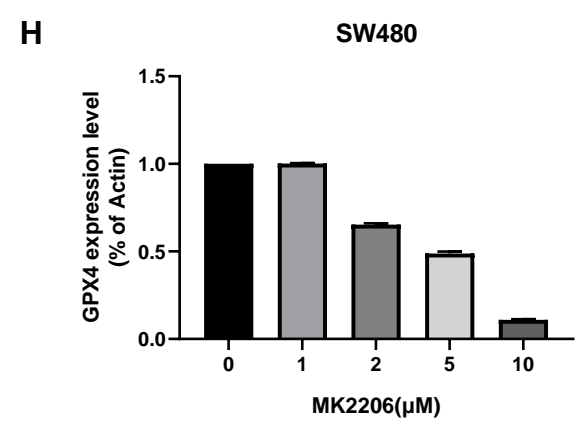

Supplement: Supplementary file 4 — Fig S1 [file 41420_2023_1746_MOESM4_ESM.pdf]

A

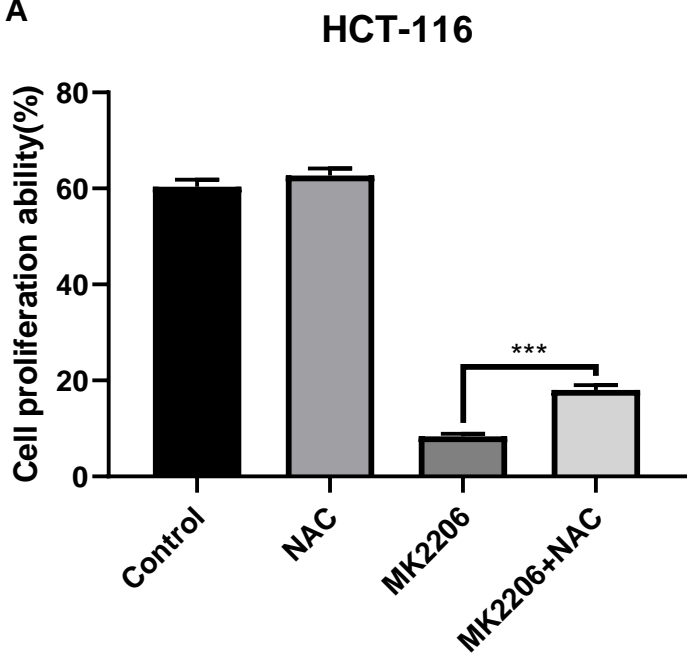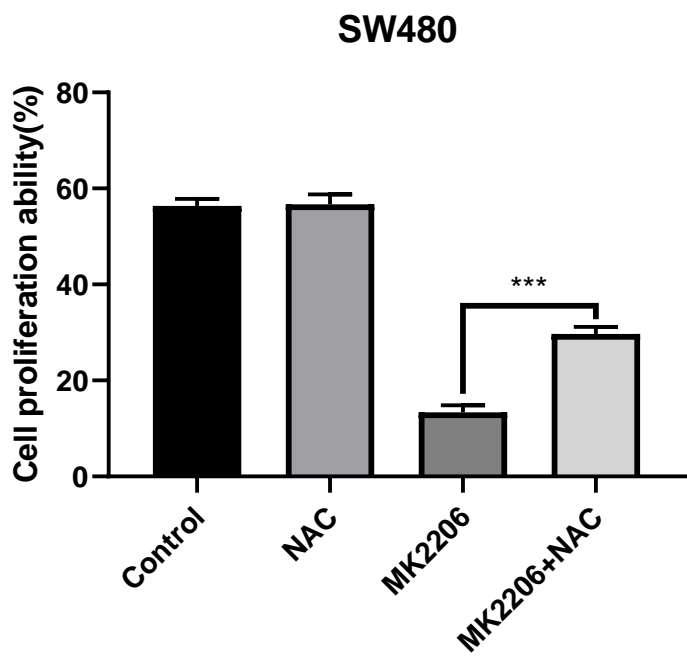

Supplement: Supplementary file 5 — Fig S2 [file 41420_2023_1746_MOESM5_ESM.pdf]

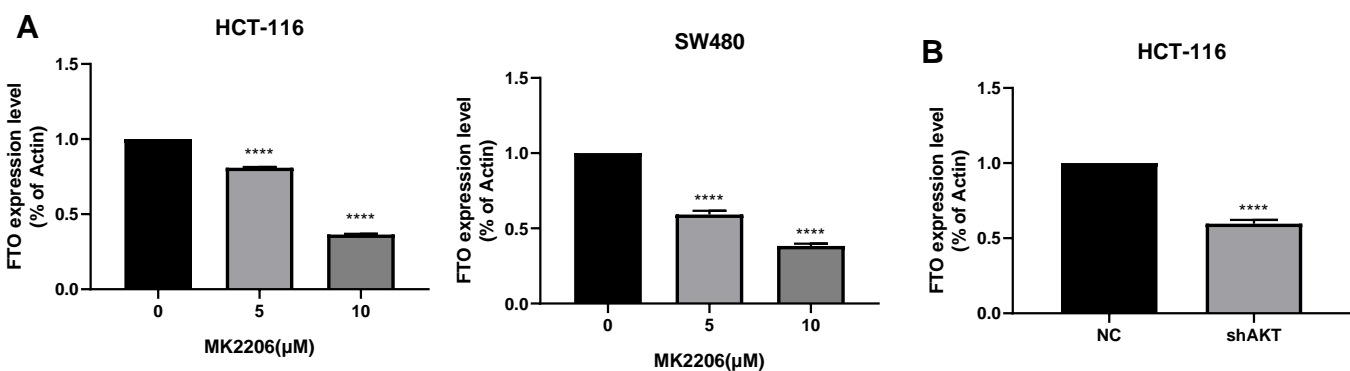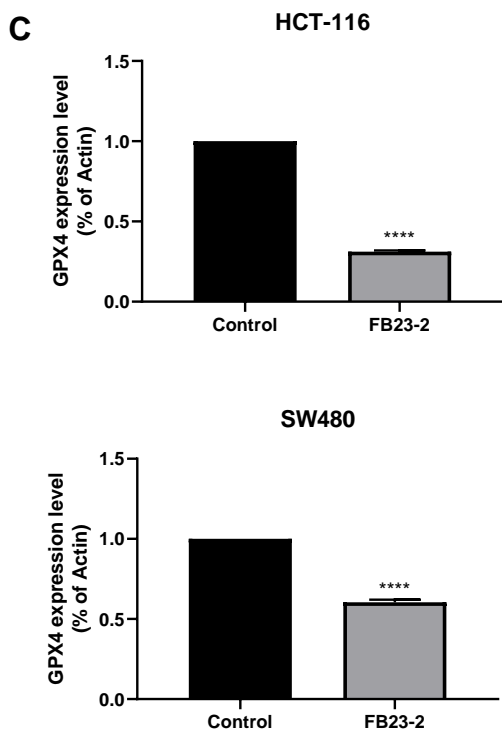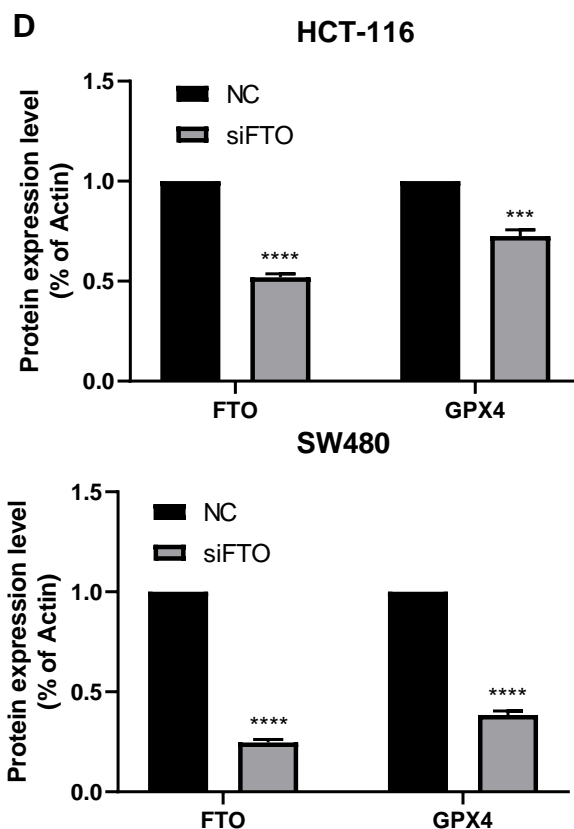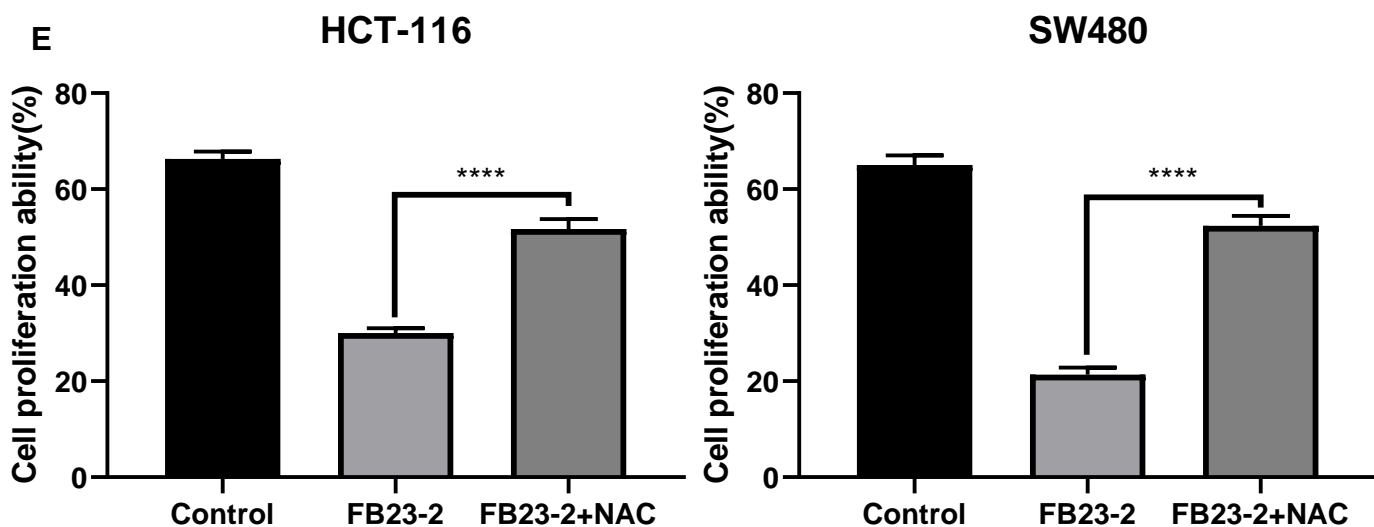

Supplement: Supplementary file 6 — Fig S3 [file 41420_2023_1746_MOESM6_ESM.pdf]

# TCGA

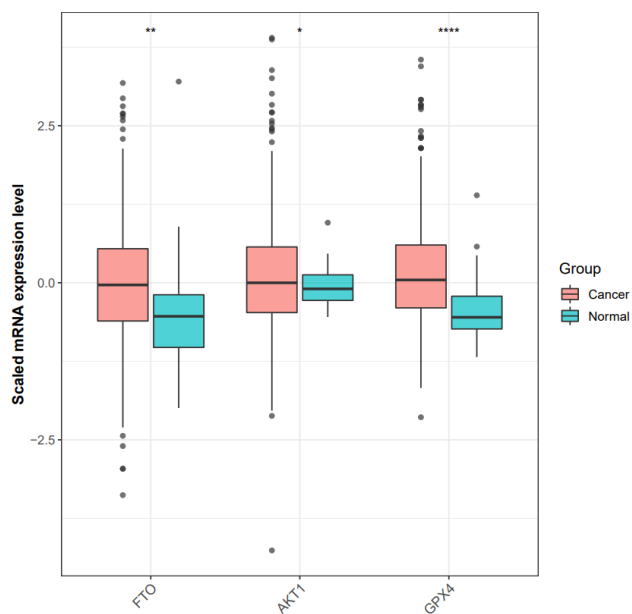

## ROC

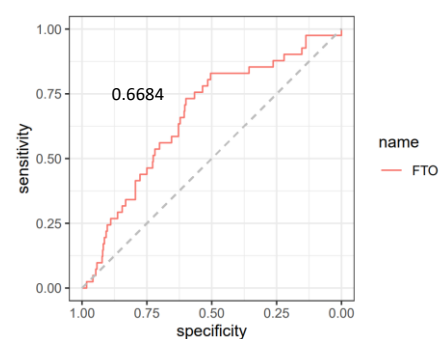

## ROC

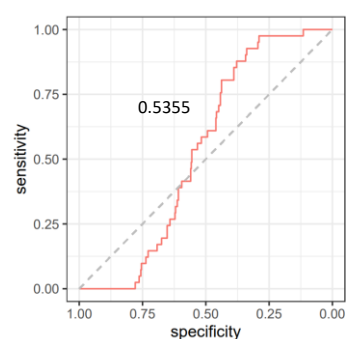

## ROC

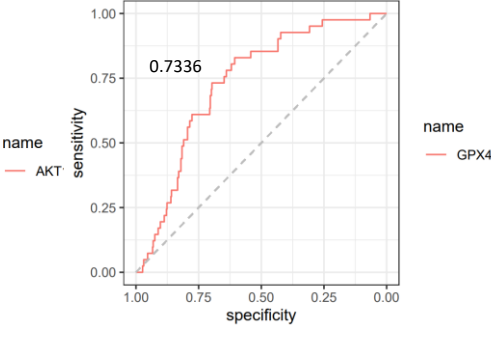

Supplement: Supplementary file 7 — Fig S4 [file 41420_2023_1746_MOESM7_ESM.pdf]

**A**

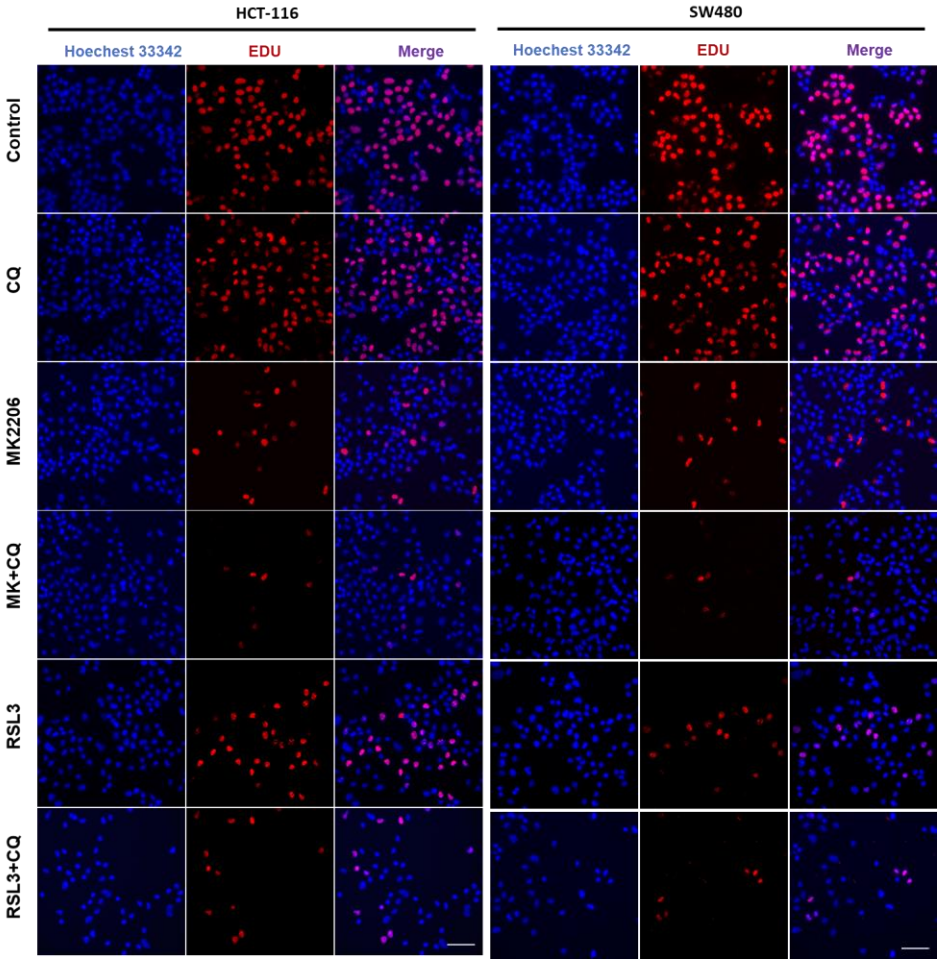

**B**

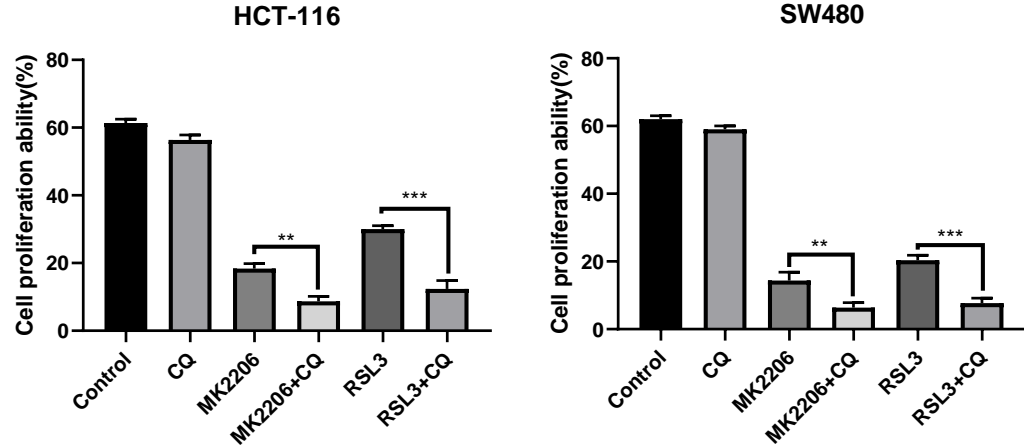

**C**

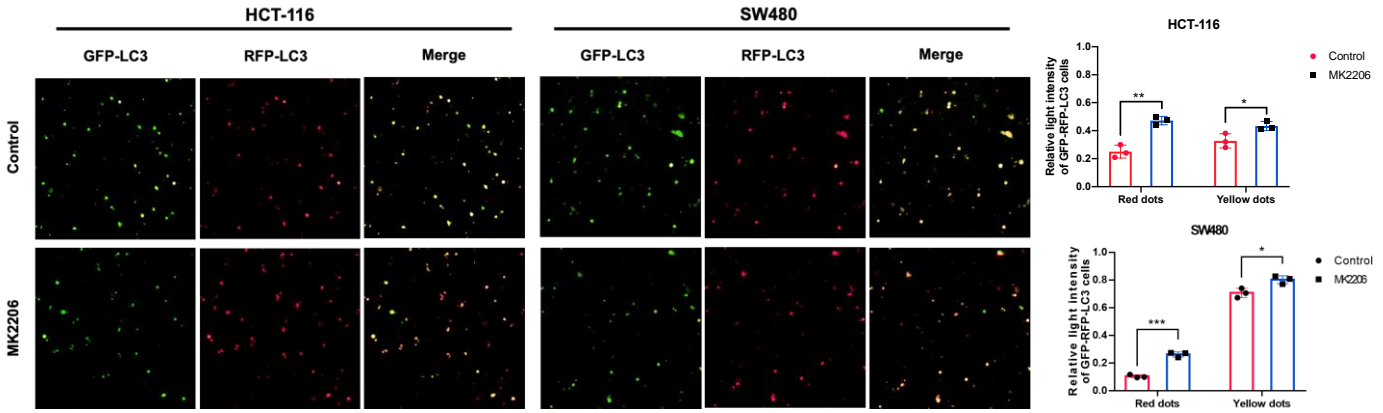

Supplement: Supplementary file 9 — Fig S6 [file 41420_2023_1746_MOESM9_ESM.pdf]
