# Supplementary material for: Targeting AKT induced Ferroptosis through FTO/YTHDF2-dependent GPX4 m6A methylation up-regulating and degradating in colorectal cancer
Source: Cell Death Discov. 2023 Dec 15;9:457. doi: 10.1038/s41420-023-01746-x (PMC10724184; doi:10.1038/s41420-023-01746-x)
Supplement: Supplementary file 3 — SUPPLEMENTAL MATERIAL Table S1 [file 41420_2023_1746_MOESM3_ESM.docx]

**FTO**

FP: GCTGCTTATTTCGGGACCTG

RP: AGCCTGGATTACCAATGAGGA

**GPX4**

FP: GGGACCATGTGCGCGT

RP: ACTTCGGTCTTGCCTCACTG

**GPX4-1**

FP: CGCCGCGATGAGCCT

RP: GTGACGATGCACACGAAGC

**GPX4-2**

FP: CACCGTCTCTCCACAGTTCC

RP: ACGCTGGATTTTCGGGTCTG

**GPX4-3**

FP: GGACCTGCCCCACTATTTCTA

RP: TTTATTCCCACAAGGTAGCCAG
