# Supplementary material for: Targeting AKT induced Ferroptosis through FTO/YTHDF2-dependent GPX4 m6A methylation up-regulating and degradating in colorectal cancer
Source: Cell Death Discov. 2023 Dec 15;9:457. doi: 10.1038/s41420-023-01746-x (PMC10724184; doi:10.1038/s41420-023-01746-x)

A

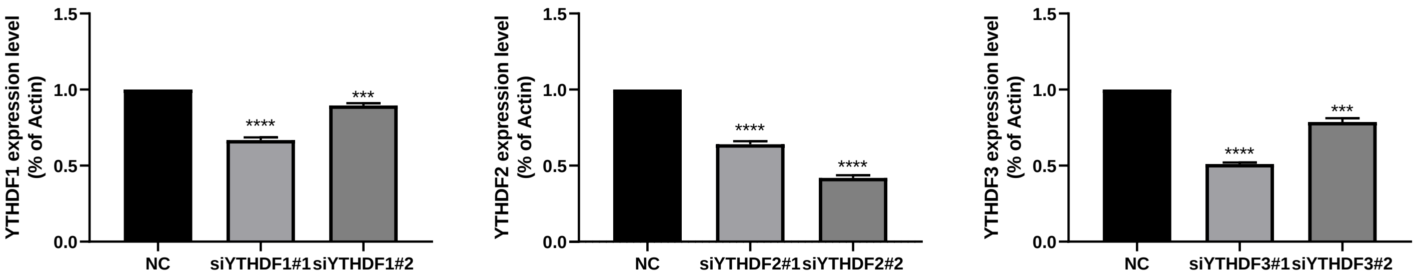

B

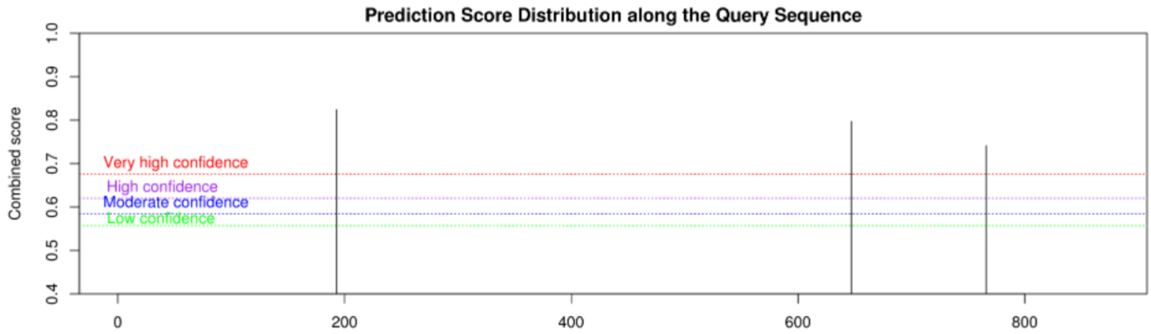

#	Position	Sequence context	Structural context	Local structure visualization	Score(binary)	Score(knn)	Score(spectrum)	Score(combined)	Decision
1	193	AUGCA CGAGU UUUCC<br>GCCAA GGACA UCGAC<br>GGGCA CAUGG UUAAC	N/A	N/A	0.846	0.708	0.808	0.824	m^6A site (**Very high** confidence)
2	647	GAGCC CCUGG UGAUA<br>GAGAA GGACC UGCCC<br>CACUA UUUCU AGCUC	N/A	N/A	0.808	0.474	0.822	0.797	m^6A site (**Very high** confidence)
3	766	UGCAA ACCUG CUGGU<br>GGGGC AGACC CGAAA<br>AUCCA GCGUG CACCC	N/A	N/A	0.752	0.240	0.789	0.741	m^6A site (**Very high** confidence)

647

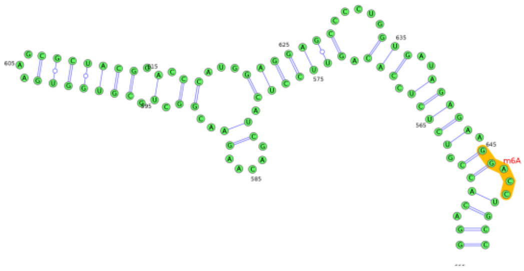

193

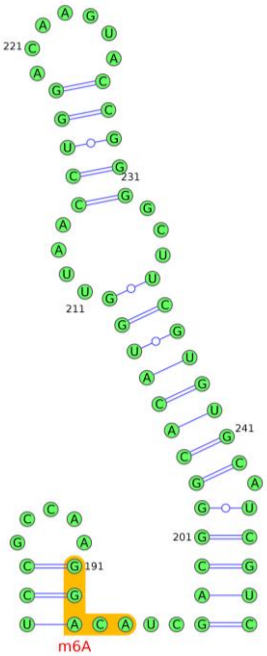

766

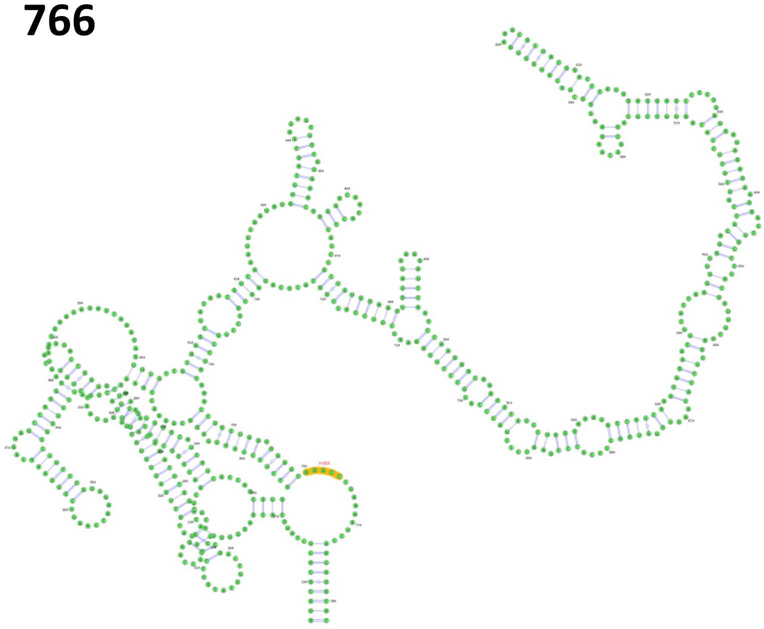

Supplement: Supplementary file 8 — Fig S5 [file 41420_2023_1746_MOESM8_ESM.pdf]
