# Supplementary material for: Targeting AKT induced Ferroptosis through FTO/YTHDF2-dependent GPX4 m6A methylation up-regulating and degradating in colorectal cancer
Source: Cell Death Discov. 2023 Dec 15;9:457. doi: 10.1038/s41420-023-01746-x (PMC10724184; doi:10.1038/s41420-023-01746-x)

Figure 1

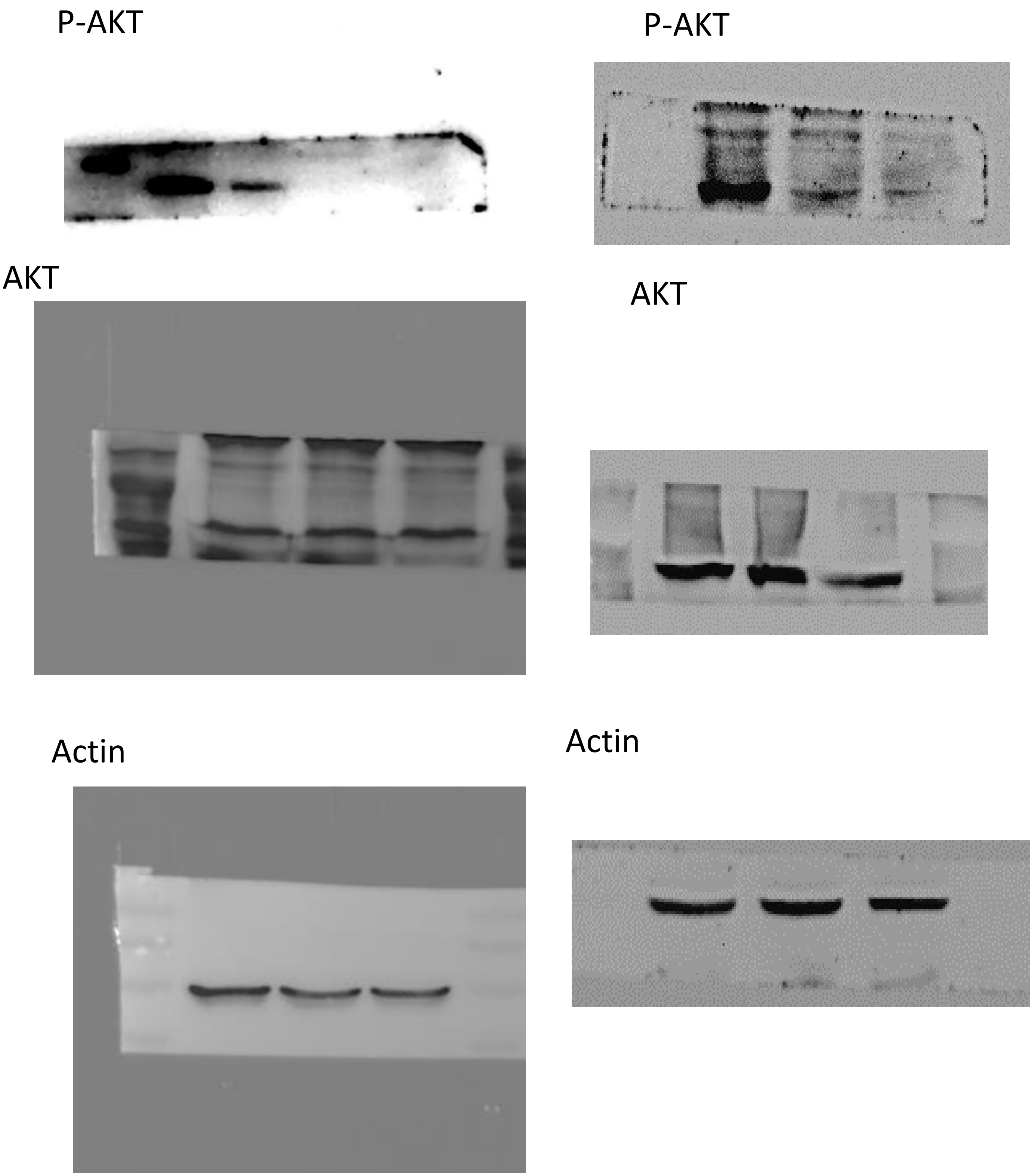

Figure s1

P-AKT

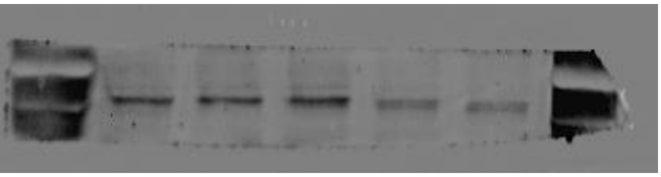

AKT

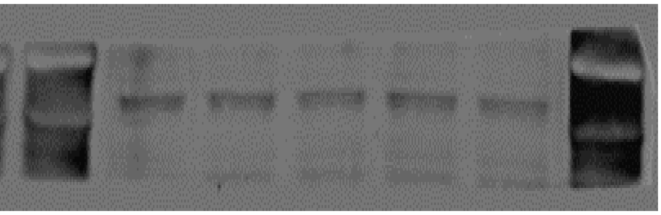

P-4EBP1

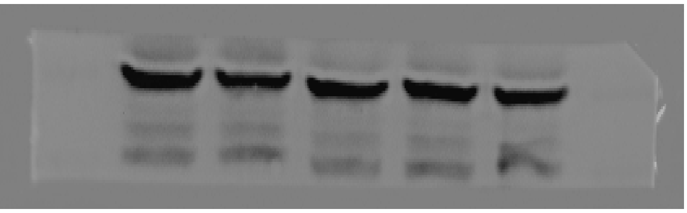

4EBP1

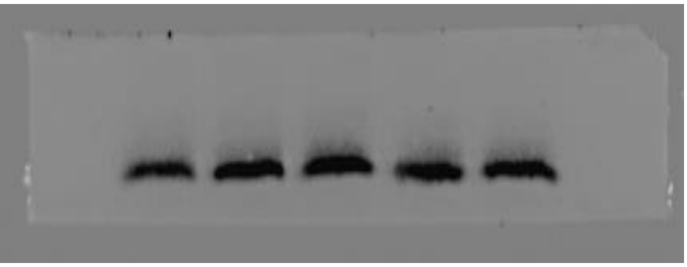

GPX4

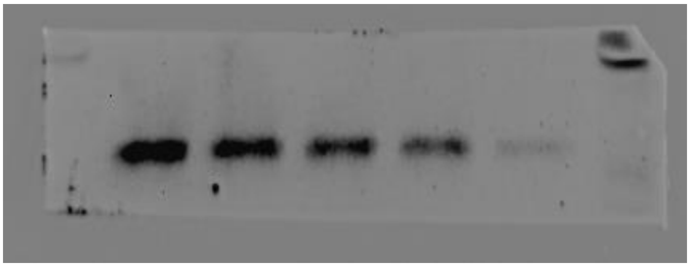

ACTIN

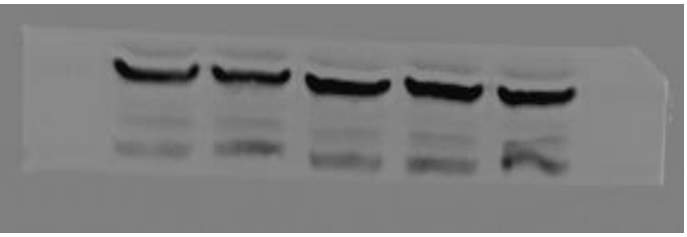

P-AKT

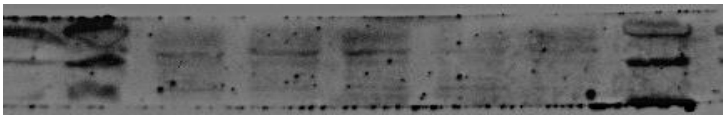

AKT

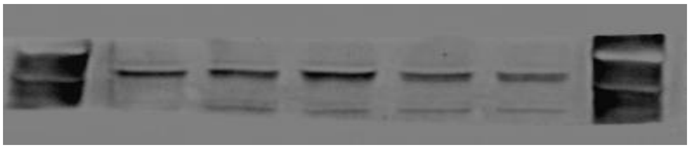

P-4EBP1

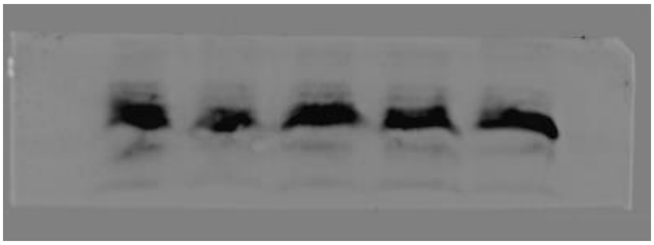

4EBP1

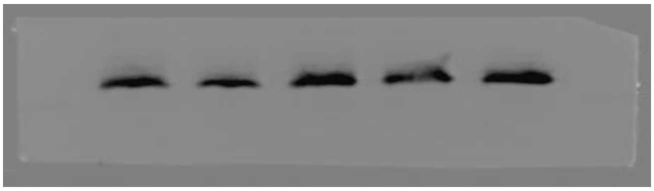

GPX4

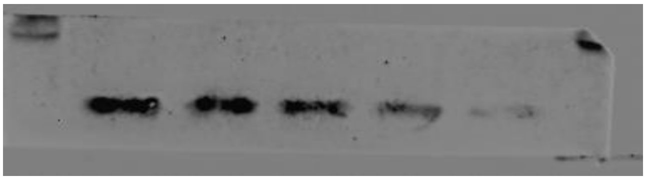

ACTIN

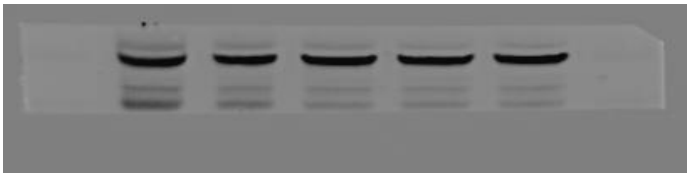

Figure 2

GPX4

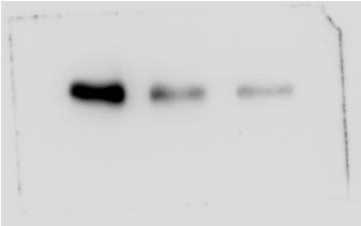

GPX4

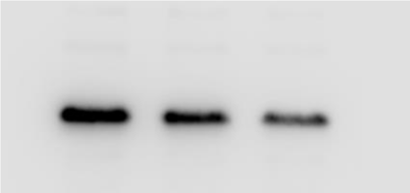

AKT

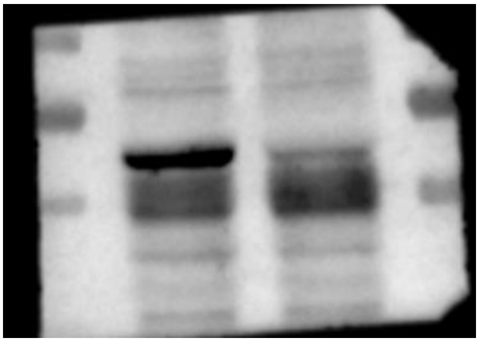

ACTIN

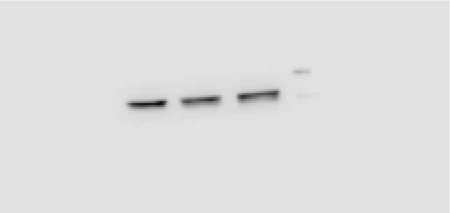

ACTIN

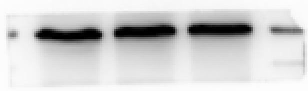

GPX4

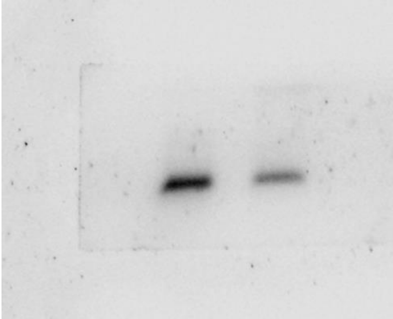

ACTIN

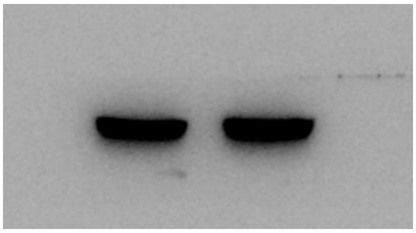

Figure 3

GPX4

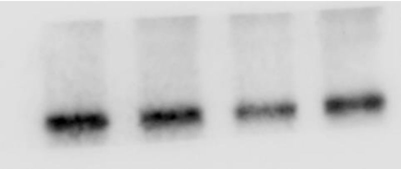

ACTIN

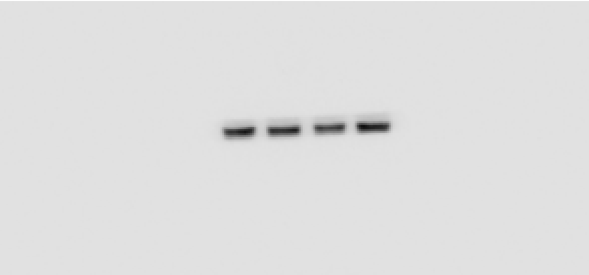

GPX4

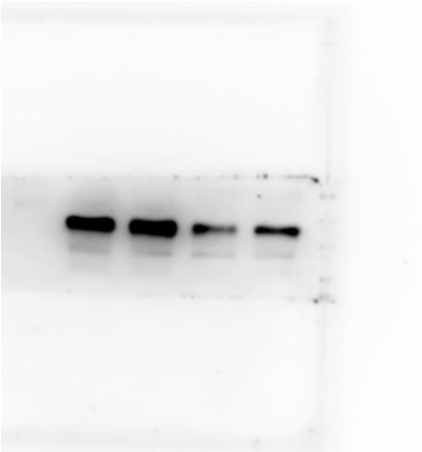

ACTIN

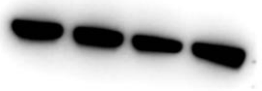

Figure 4

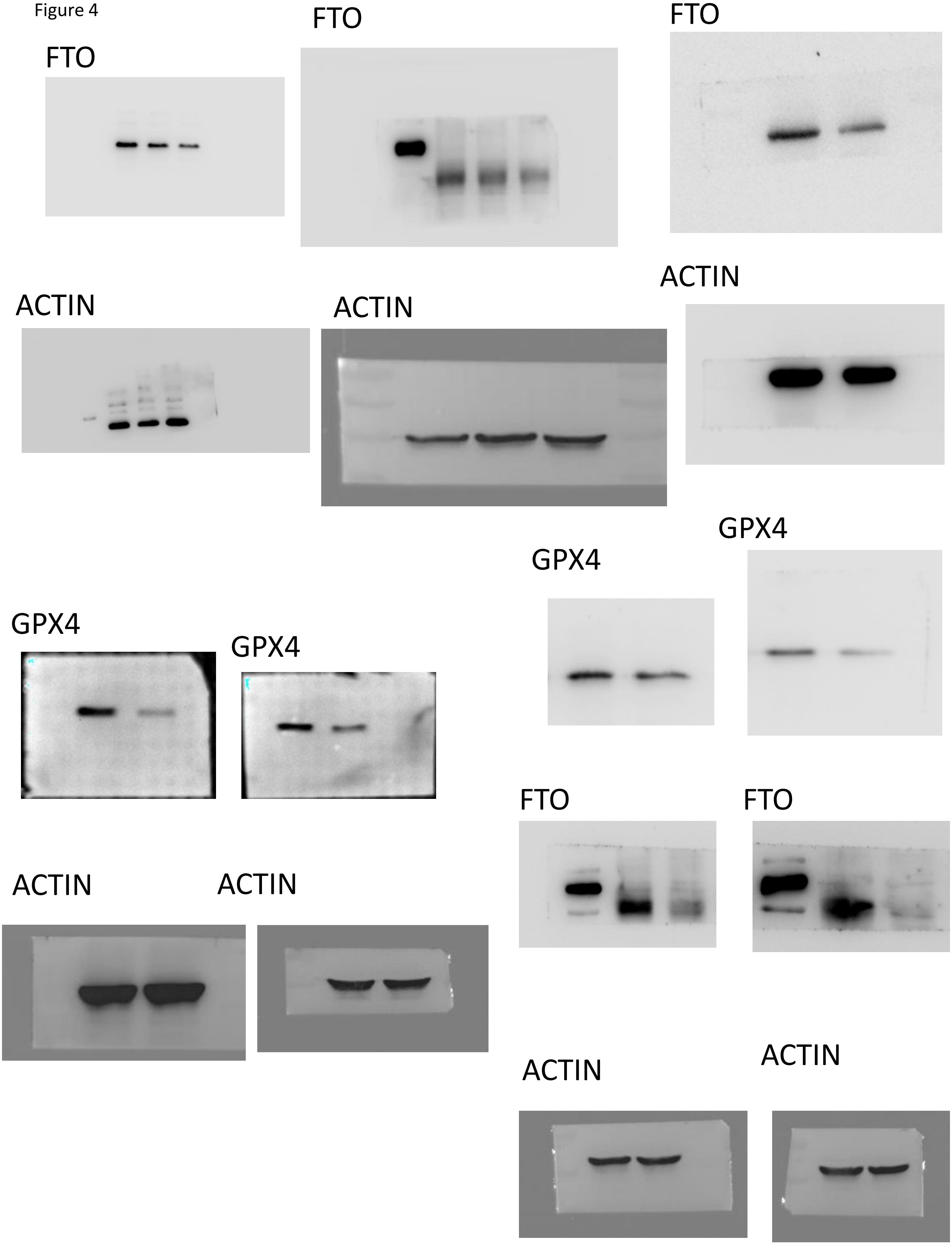
FTO
FTO
FTO
ACTIN
ACTIN
ACTIN
GPX4
GPX4
GPX4
GPX4
FTO
FTO
ACTIN
ACTIN
ACTIN
ACTIN

Figure 5

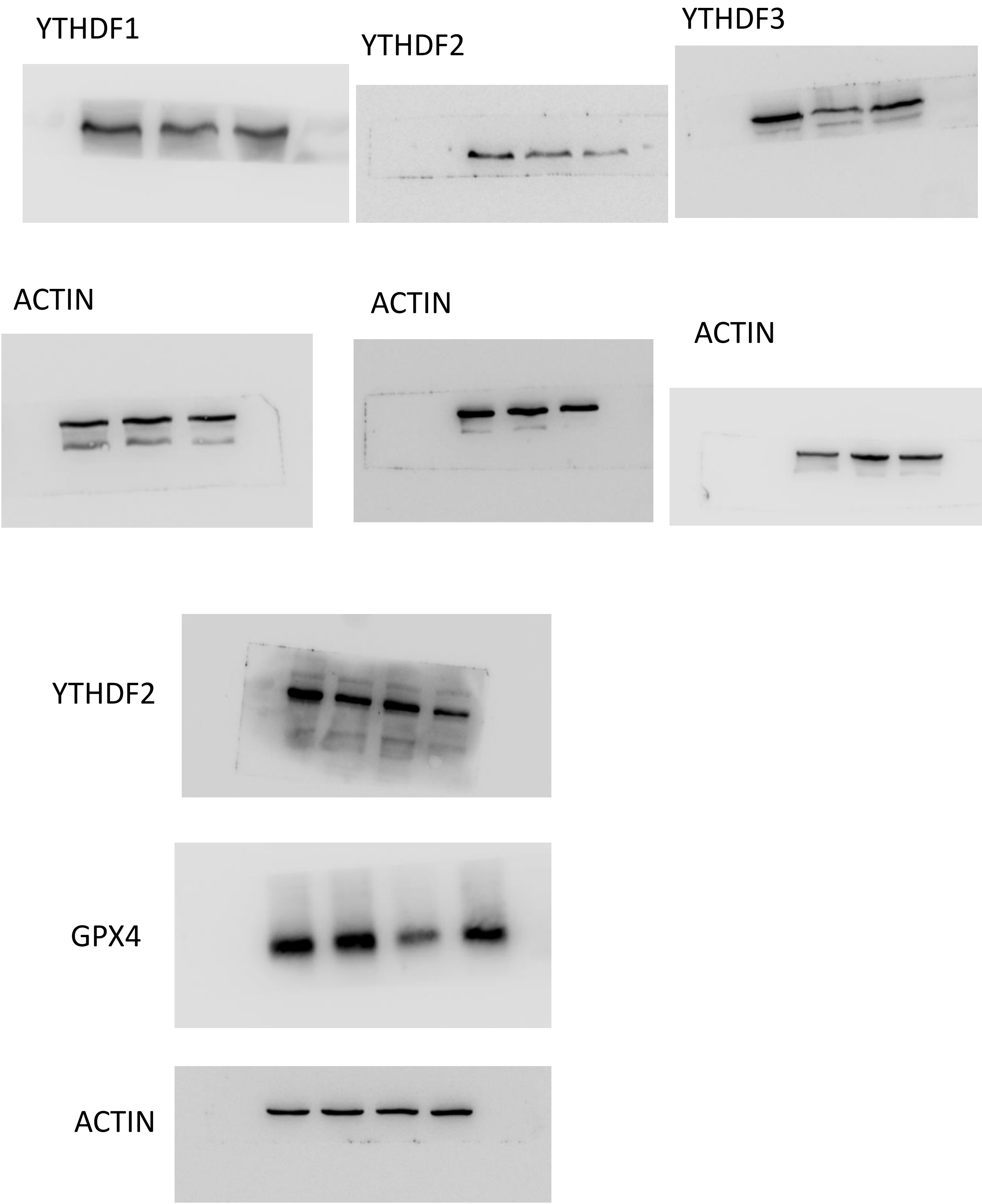

Figure

LC3

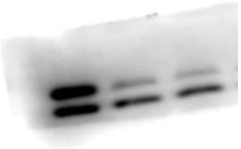

ACTIN

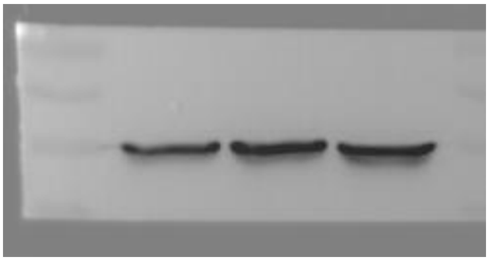

GPX4

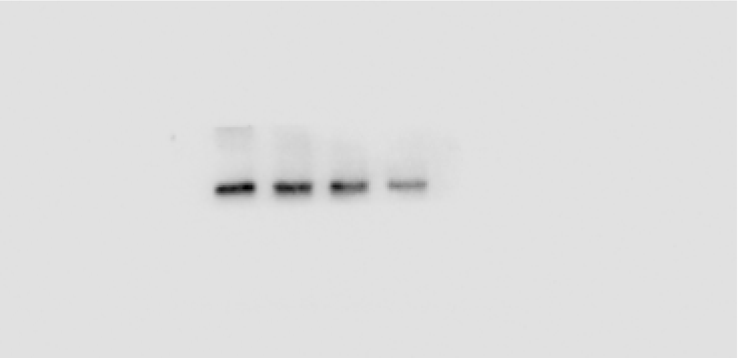

ACTIN

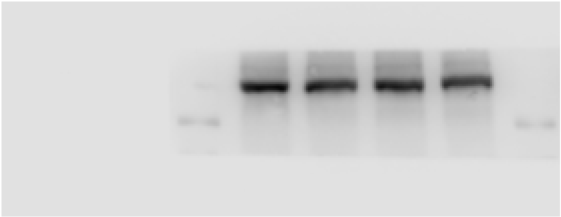

LC3

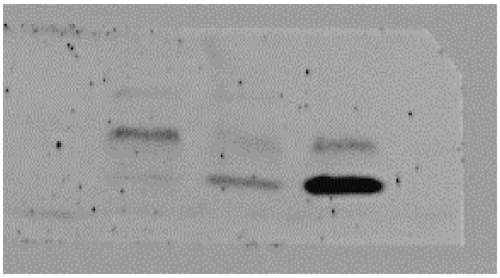

ACTIN

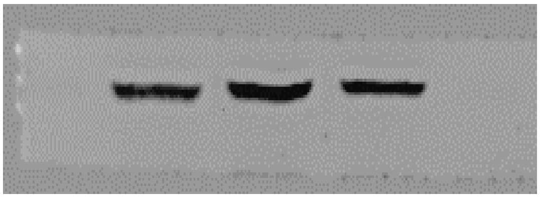

GPX4

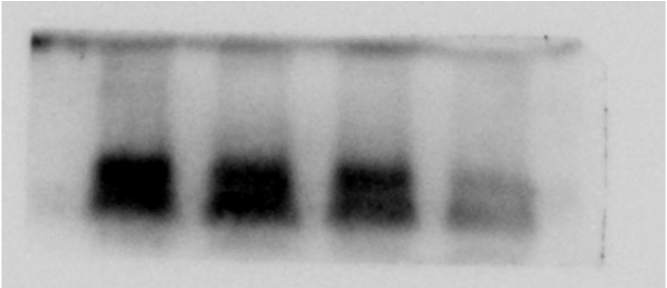

ACTIN

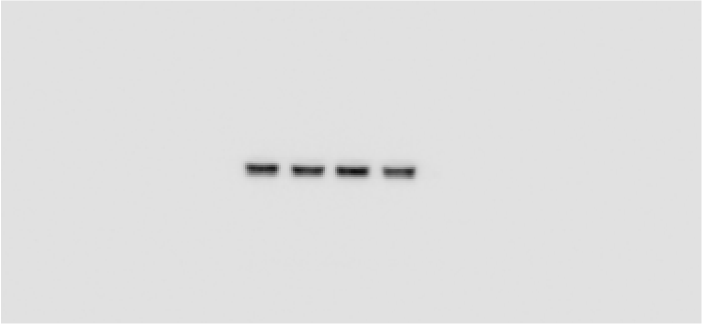

Supplement: Supplementary file 10 — Initial western blotting [file 41420_2023_1746_MOESM10_ESM.pdf]
